# Transection of the Superior Sagittal Sinus Enables Bilateral Access to the Rodent Midline Brain Structures

**DOI:** 10.1523/ENEURO.0146-21.2021

**Published:** 2021-07-14

**Authors:** Marcelo Dias, Inês Marques-Morgado, Joana E. Coelho, Pedro Ruivo, Luísa V. Lopes, Miguel Remondes

**Affiliations:** Instituto de Medicina Molecular João Lobo Antunes (IMM-JLA), Faculdade de Medicina, Universidade de Lisboa, Lisbon 1649-028, Portugal

**Keywords:** superior sagittal sinus, surgery

## Abstract

Stereotaxic access to brain areas underneath the superior sagittal sinus (SSS) is notoriously challenging. As a major drainage vessel, covering the whole extension of the sagittal fissure, the SSS impedes direct bilateral access to underlying regions for recording and stimulation probes, drug-delivery cannulas, and injection devices. We now describe a new method for transection and retraction of the SSS in rats, that allows the accurate placement of microinjection devices, or chronic electrode probes, while avoiding hemorrhage and the ensuing deleterious consequences for local structures, animal health, and behavior. To demonstrate the feasibility of this approach we evaluated its consequences acutely during surgery, and thereafter during surgical survival, recovery, behavioral testing, as well as postmortem analysis of histologic impact in the related brain structures of male rats. This method provides a new approach enabling direct access for manipulation and recording of activity in brain areas previously obstructed by the SSS.

## Visual Abstract

**Figure:**
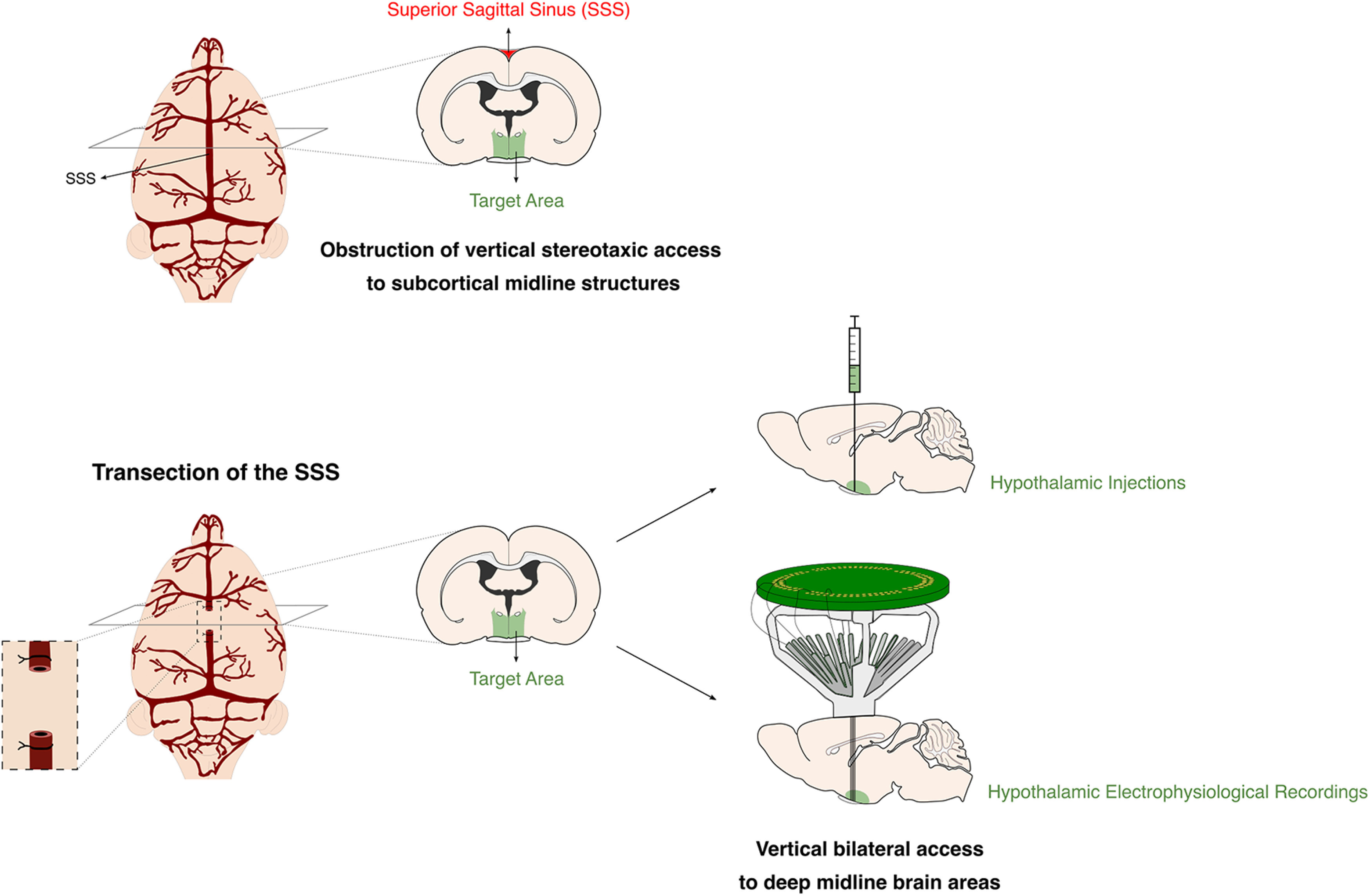

## Significance Statement

The superior sagittal sinus (SSS) constitutes a physical obstacle for the vertical bilateral stereotaxic access to cortical and subcortical midline structures, often leading to extensive bleeding, damage to the underlying brain tissue and animal death. To overcome these frequent practical difficulties, we have developed and validated an innovative surgical method to transect and retract a small portion of the SSS, exposing the sagittal fissure with negligible vascular or neural damage. Transection of the SSS provides a new approach for manipulation and recording of activity in directly inaccessible midline brain regions, preserving cognitive, physiological, and anatomic integrity.

## Introduction

Recording from and manipulating populations of neurons in target brain regions is fundamental for understanding neural function (Cui et al., 2013; Jun et al., 2017; Liang et al., 2017; Kampasi et al., 2018; Pisanello et al., 2018; Steinmetz et al., 2018; Luo et al., 2020). However, bilateral stereotaxic access to cortical and subcortical midline structures has been notoriously difficult because of the obstruction of vertical approach paths by the overlaying superior sagittal sinus (SSS). Surgical manipulation of the SSS or insertion of probes in close proximity often leads to ruptures and extensive bleeding, consequently damaging the underlying brain tissue with critical implications for neural function. Bleeding from injuries to the SSS frequently causes accumulation of blood under the meninges, increasing intracranial pressure (Jussen et al., 2017) and inflammation (Quan et al., 2015), and causing ischemic neuronal damage to the underlying cortex (Chen et al., 1991). This damage often extends beyond the underlying cortex, affecting subcortical structures, such as the hippocampus (Jussen et al., 2017). Moreover, the consequences of acute subdural hematomas are frequently more harmful than expected from simple increases in intracranial pressure, with evidence suggesting that the presence of blood constituents aggravates histologic and functional damage (Sasaki and Dunn, 2001; Baechli et al., 2010; Jussen et al., 2017).

It is thus critical to avoid damage and bleeding from the SSS to preserve tissue viability. One technique to avoid or minimize such damage is to target deep midline structures at an angle from a lateral coordinate, instead of directly from above (i.e., vertically). This method has the advantage of being relatively simple and allowing potentially damaging probes or instruments, like electrodes or sharp pipettes, to be kept away from the SSS. However, angled approaches are problematic for reaching deeper structures, such as small midline hypothalamic nuclei, where minor deviations from the intended path lead to gross mistargeting. Additionally, in the case of particular targets (e.g., hypothalamic nuclei), an angled approach is suboptimal for *in vivo* electrophysiological recordings (Jun et al., 2017; Liang et al., 2017), as depth adjustments and probe designs lead electrodes to cross target structures through a plane of reduced thickness (i.e., orthogonal to the major axis of the region), thus minimizing the number of recorded units per penetration. A second, commonly employed approach, is to dislocate the sinus laterally to expose the target mediolateral coordinate (Wirtshafter et al., 1979). This approach has recently been used to implant optical recording devices for prefrontal cortex and medial entorhinal cortex imaging in head-fixed animals (Low et al., 2014). However, bulky tetrode drives and sensitive silicone probes preclude the simultaneous mobilization of the SSS, and the lowering of probes in the confined space of a craniotomy without increased risks of damage to either. Furthermore, laterally dislocating the SSS only exposes one hemisphere and simultaneous bilateral access is still impeded. Importantly, mobilization per se can lead to injury and bleeding (personal observations), and implanting unilateral probes in close contact to the SSS can cause ruptures at a later point in time if probes need to be lowered postsurgically to sample a new pool of units (e.g., silicone probes).

Direct, unobstructed, vertical access solves many of the challenges inherent to bilateral stereotaxic targeting of deep midline brain regions. Using simplified approach paths would greatly improve the positioning of the recording and stimulation devices, and minimize surgical complications resulting from SSS damage. Here, we report a two-step surgical procedure to transect and retract the SSS, enabling direct bilateral access to the underlying cortex and the longitudinal fissure. First, the SSS is double ligated rostrally and caudally in relation to the target anteroposterior coordinates. Next, the SSS is cauterized between the ligature sites and retracted to expose the underlying tissue. Using the method detailed in this protocol, we have successfully implanted chronic octrode drives and injected neural tracers and viral vectors in anterior and intermediate hypothalamic nuclei, with negligible vascular or neural damage. Moreover, we found that behavioral parameters and histologic markers were comparable to those of sham-operated animals, confirming that this method is a viable approach to gain vertical bilateral access to deep midline brain areas without compromising behavioral outcomes.

## Materials and Methods

### Subjects

A total of 28 male rats (27 Sprague Dawley and one Long–Evans; *Rattus norvegicus*) were used for all procedures. Animals were two to three months old, 350 and 480 g body weight (Charles River Laboratories) at the time of the surgeries. One animal died during the surgery.

Rats were housed in groups of four to five per ventilated double-decker cage and kept under constant experimental conditions: 14/10 h light/dark cycle, 21 ± 0.5°C, 60 ± 10% relative humidity, food and water *ad libitum*.

All the procedures were approved by the Portuguese National Authority for Animal Health (DGAV), as well as by the institute’s animal well-being office.

#### Surgical procedures

#### Transection of SSS

Materials and step-by-step surgical procedure are described in Detailed Protocol (https://imm.medicina.ulisboa.pt/intranet/links/view_file/TAS66TLXI/2633).

In control animals, without SSS transection, all surgical steps were equal to the experimental group, except the ligation and sectioning of the sinus. In the sham surgery, the suture was threaded under the sinus as described above and removed shortly after, without ligation.

#### Microinjection of virus and tracer

After SSS transection was performed, animals were injected either with a viral construct (AAV8-hSyn-hM4D(Gi)-mCherry) or an anterograde neural tracer [biotinylated dextran amine (BDA)–Texas Red conjugated], using a microinjection control system attached to the stereotaxic frame and a glass micropipette.

The micropipette was filled with mineral oil and placed in the microinjector, after which the virus or tracer were aspirated to fill the tip of the pipette.

Using bregma as a reference, the pipette was carefully positioned over the microinjection coordinates, slowly lowered until reaching the targets [medial preoptic area (MPOA): −0.60 mm AP, +/−0.3 mm ML, −8.4 mm DV; suprachiasmatic nucleus (SCN): −0.60 mm AP, +/−0.3 mm ML, −9.2 mm DV] and left in place for 5 min. The virus/tracer was then delivered (0.5 μl in MPOA or 0.1 μl in SCN), at a rate of 100 nl/min. Micropipette was slowly retracted after waiting 10 min, to avoid contamination of overlaying structures.

Craniotomy was then covered with a single drop of 1.5% agarose at body temperature (∼37°C). Skin incision was sutured and Ringer’s lactate was administered for hydration. For postoperative recovery, the animals were placed in a heated cage during the first 24 h. During this period, *ad libitum* food, nutritional gel, and water were provided.

#### Hyperdrive implant

After exposing the skull and identifying stereotaxic landmarks, seven anchor screws were fixed to the skull. An additional screw was placed over the cerebellum to serve as ground. The craniotomy (AP: 1 to −3, ML: 2 to −2) was carefully opened and the SSS transection performed as described above. Mineral oil was then applied to the surface of the brain, and the hyperdrive was carefully lowered and firmly fixed to the skull and to the anchor screws with self-curing dental acrylic (Kerr Tab 2000).

The hyperdrive (adapted from Liang et al., 2017) carried 15 independent octrodes each consisting of eight 12.7-μm nichrome wires (Sandvik). The distance between octrodes was ∼166 μm. A single rectangular octrode bundle (1.46 × 1.27 mm, including the surrounding epoxy used to fix the polyamide tubes) was implanted to bilaterally target the hypothalamus at AP: −0.33 to −1.08, ML: 0.5 to −0.5 and DV: −9.2.

Once the dental acrylic hardened, the octrodes were manually lowered to the desired depth and glued in place. We attached a small “ball” of super glue at the desired octrode length (above the upper end of the polyamide tubes) to serve as a bumper, effectively allowing octrodes to be manually lowered to their target depths. This lowering movement was smooth and unimpeded and we did not observe blood exiting the polyamide tubes. Octrodes were fixed throughout the recording experiment.

The surgical wound was closed, Ringer’s lactate (5 ml) and carprofen (5 mg/kg) were administered subcutaneously, and the animal was left to recover over the period of one week. To prevent infections, the animal received minocycline diluted in the drinking water (100 mg/l) throughout the experiment.

The postmortem extraction of the probe was performed by first ventrally exposing the brain and gently freeing it from the surrounding skull. Octrodes were found to be straight and the hyperdrive was retrieved after careful removal of bone and dental acrylic remains. Although aiming at simultaneous bilateral recordings, postmortem histologic verification revealed unilateral targeting and final coordinates more posterior than expected, most likely because of unleveled drive and octrode insertion during the surgery.

### Behavioral testing

To assess potential effects of the sinus sectioning on animals’ locomotor and memory performances, all animals were tested before the surgery, as well as four and eight weeks postsurgery. Rats were handled for 5 d before behavioral tests. After basal behavioral assessment, animals were randomized between control and experimental groups. All behavioral tests were performed in a sound attenuated room, during the light phase of the circadian cycle, between 9 A.M. [zeitgeber time (ZT)2] and 7 P.M. (ZT12).

#### Open-field test

Locomotor and exploratory behavior were measured as routinely performed in the lab. The test consisted in free exploration of an arena (40 × 40 × 40 cm) for 5 min. Activity was recorded and analyzed using a video-tracking software (Smart 2.5, PanLab) to assess total distance traveled, average speed, resting time and permanence time in peripheral versus central portions of the arena.

#### Y-maze test

Short-term reference memory was assessed in a spontaneous novelty-based spatial preference Y-maze test. The test was performed as a two-trial recognition test in a Y-shaped maze with three arms (each with 35 ×10 × 20 cm), angled at 120° and with opaque walls. Different visual cues were placed at the end of each arm. Allocation of arms was counterbalanced. During the habituation phase (learning trial) rats were placed at the end of the “start” arm, allowed to explore two arms of the maze for 8 min and returned to the home cage. Access to the third arm (“novel” arm) was blocked by an opaque door. After 1 h, the door of the novel arm was removed and animals were placed again in the start arm to freely explore the maze for 5 min (test trial). Rat tracings were recorded using SMART video-tracking software. Preference for the novel arm is considered a measure of short-term reference memory.

### SHIRPA

SHIRPA is a three-stage protocol for the comprehensive analysis of behavioral phenotype of mice and rats (Rogers et al., 1997).

In order to assess possible adverse side effects of the SSS transection on general behavior phenotype that could be indicative of extensive neural damage, we resorted to the first stage of SHIRPA protocol. This is an adaptation of the Irwin procedure (Irwin, 1968) that consists of standard methods to provide a behavioral and functional profile by observational assessment. The screening of each animal begins by observing undisturbed behavior, looking for manifestation of bizarre or stereotypical behaviors, defects in gait, posture, motor control, coordination, and breathing rate, as well as alterations in coat appearance, piloerection, palpebral closure, lacrimation, and tremor. The observation of spontaneous behavior is followed by a sequence of manipulations, with tail suspension and use of the grid across the width of the cage, to assess positional passivity, limb grasping, irritability, aggression, and visual perception.

Evaluated parameters are scored to provide qualitative assessment of functional phenotype with gross measures of sensory, spinocerebellar, neuropsychiatric, autonomic, and motor functions.

### Histology

#### Pathology

A total of 10 weeks after SSS transection, rats were euthanized with Isofluorane overdose and immediately decapitated. Whole heads were immersion fixed in 10% neutral buffered formalin, followed by 72 h in a decalcifying solution (Surgipath Decalcifier Leica), and then routinely processed for paraffin embedding, sectioned at 4 μm (15–20 sections distancing 100 μm), and stained with hematoxylin and eosin. Lesions were classified by a veterinary pathologist blind to the experimental groups, and scored according to the severity: absent, minimal, mild, moderate, marked, and severe.

To quantify the histologic damage, lesioned area was measured in five serial sections per animal (across 1.5 mm) centered in the zone where the transection was performed and normalized to the whole-brain area.

All slides were digitally scanned using Hamamatsu NanoZoomerSQ, and representative images were obtained using NDP.view2 software (Hamamatsu).

#### Anatomical tracing

Five weeks (viral vector) or 11 d (BDA) postinjection, animals were euthanized as above and transcardially perfused with ∼300 ml of PBS followed by ∼500 ml of 10% neutral buffered formalin. Brains were gently dissected from the skull to avoid damage to the optic chiasm and SCN, kept in formalin for 24 h at room temperature, then placed in 15% sucrose in PBS until sinking, followed by 30% sucrose in PBS. After sinking, the brains were embedded in gelatin and frozen. Frozen brains were sectioned coronally in 50-μm slices using a cryostat (LEICA, CM3050 S). Fixed brain slices were de-gelatinized in PBS at 37°C for 10 min, mounted in Fluoromount, coverslipped, and left to dry in a dark place. The virus/tracer expression was confirmed using an Axio Observer widefield fluorescence microscope (Zeiss) equipped with an Axiocam 506 mono CCD (Zeiss).

#### Octrode tracks

Four weeks after the hyperdrive implant, the animal was euthanized and perfused. The brain was processed as above, and slices were incubated for 10 min with Hoechst (Hoechst 33342, Thermo Scientific, 12-μg/ml final concentration), washed for 15 min with PBS, mounted in Fluoromount, cover-slipped and left to dry in a dark place. Octrode locations were confirmed using an Axio Observer widefield fluorescence microscope (Zeiss) equipped with an Axiocam 506 mono CCD (Zeiss).

### Analyses

#### Analysis of electrophysiological recordings from hypothalamic nuclei

Analysis of *in vivo* electrophysiological signals was performed using the Spikeinterface toolbox (Buccino et al., 2020) together with custom written Python and MATLAB (MathWorks) scripts. Raw data were bandpass filtered between 300 and 6000 Hz and spiking activity was extracted based on four SD threshold crossings. Spike sorting was performed using mountainsort4 (Chung et al., 2017).

#### Statistical analysis

All statistical analyses were performed with GraphPad Prism software. Values are presented as dot blots with individual values plus bar with mean ± SEM. Statistical comparisons included two-sided unpaired *t* test, repeated measures one-way or two-way ANOVA, and two-way ANOVA, followed by a Bonferroni’s or Tukey’s multiple comparison *post hoc* tests, as specified in the figure legends; *p* < 0.05 was considered to be statistically significant.

### Materials

Please see detailed protocol https://imm.medicina.ulisboa.pt/intranet/links/view_file/TAS66TLXI/2633

## Results

### Transection of the SSS, overview

We developed a two-step method for safely transecting the SSS with minimal damage to the surrounding vasculature or brain tissue (detailed protocol and materials available at https://imm.medicina.ulisboa.pt/intranet/links/view_file/TAS66TLXI/2633 and minimal increase in surgery time or surgical complexity (∼124 min from anesthesia to wound closure in ST animals, as compared with 86 min in sham animals).

**Figure 1.**
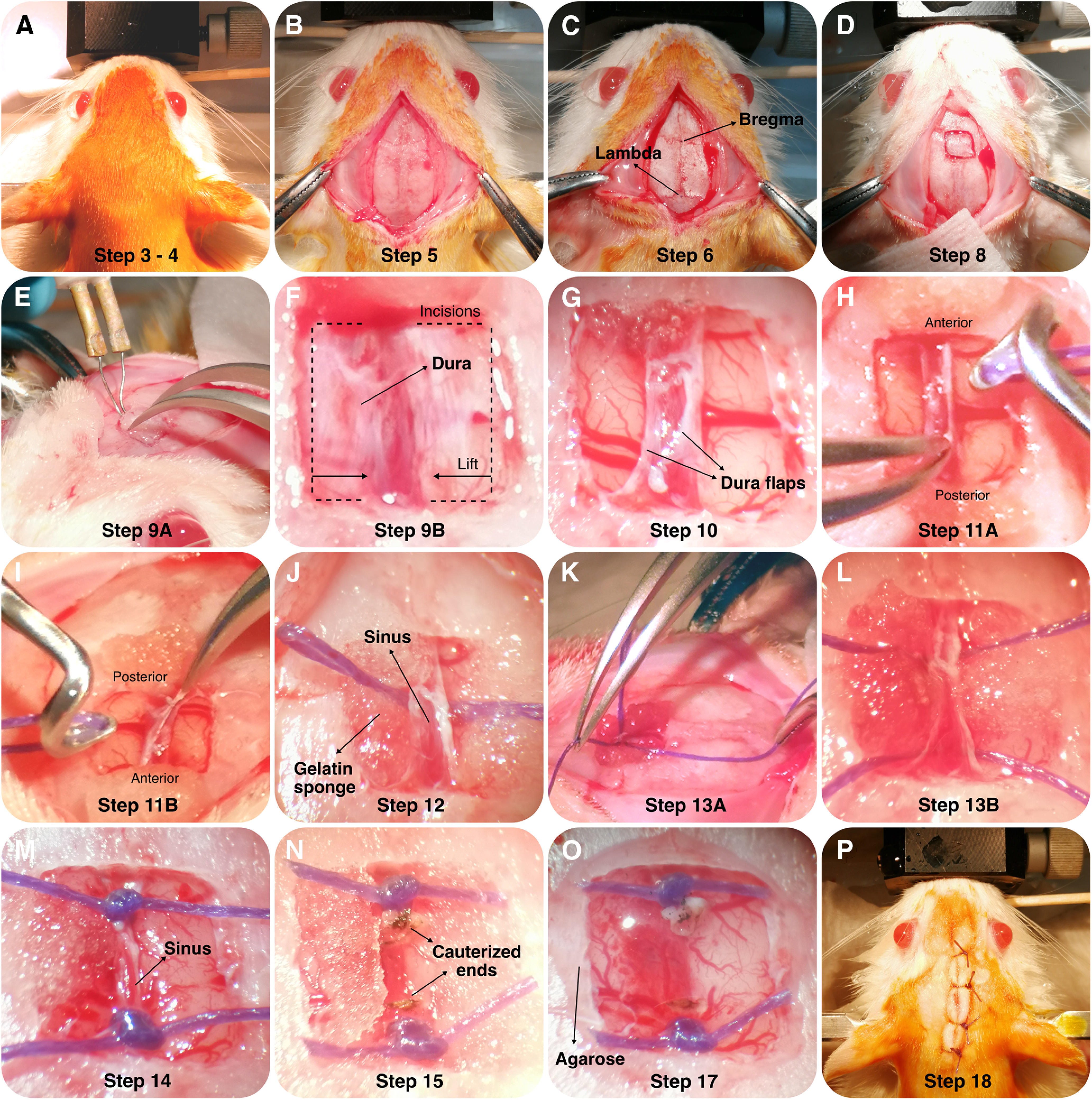
Detailed photographs of the surgical procedure. ***A***, Induce anesthesia and verify the absence of reflexes. After careful fixation to the stereotaxic frame, scrub the surgical site with antiseptic to avoid contamination. ***B***, Make an incision along the sagittal fissure. Expose the skull by scrapping periosteum off the bone. ***C***, Identify the stereotaxic landmarks (Bregma and Lambda) using the sutures on the skull surface. Validate horizontality of the skull. ***D***, Mark the outline of the craniotomy on the skull surface. ***E***, Lift the single bone piece using a double hand technique.Use forceps on one hand to slowly lift the bone flap and a cauterizer with the smallest tip attached on the other hand to scrape the dura of the bone as it is gradually lifted. ***F***, Open each durotomy at the lateral limit of the craniotomy, next to the bone, and extend it carefully along the anterior and posterior boundaries of the craniotomy until the border of the sagittal sinus. ***G***, Slowly lift and fold the bilateral dura flaps over the sinus. ***H***, Elevate the attached sinus and thread a suture beneath it, using a vascular ligation instrument. ***I***, Use one hand to lift the sinus and the other to slide the tip of the instrument under the sinus, carefully avoiding cerebral veins. Once the instrument’s tip is visible on the opposite site, lower the sinus and carefully thread the suture under it without removing the instrument, stabilizing it very carefully. ***J***, Once a 2-3cm loop of suture is threaded, carefully retract the instrument while firmly holding the suture in place at the opposite side of the sinus. ***K***, ***L***, Cut the loop in the middle leaving the suture in place, crossing underneath the sinus, with two strands of thread perpendicular to the sinus. Gently pull one strand towards the anterior limit of the craniotomy and the second towards the posterior limit. ***M***, Double ligate the one at the posterior border, followed shortly by the anterior one. ***N***, Gently lifting the sinus with one hand, cauterize the mid-point between sutures and extend the severed borders of the sinus posteriorly and anteriorly, exposing the longitudinal fissure. ***O***, Cover the whole extension of the craniotomy with a single drop of 1.5% agarose at body temperature (∼37°C) to mechanically stabilize the remaining sinus edges and the suture knots left on them. ***P***, Suture the skin incision, sterilize the wound and apply lidocaine analgesic ointment. Revert anesthesia.

Briefly, rats are anaesthetized and securely placed on a stereotaxic frame (Fig. 1*A*). After exposing the skull, bregma and λ are identified and used to level the animal’s head (Fig. 1*B*,*C*). Using a small drill-bit (Fine Science Tools, catalog #19007-05) the craniotomy window is gradually and carefully opened until a single bone flap is loosened (Fig. 1*D*). The flap is lifted on one side with fine tweezers and with the help of a small, blunt instrument (e.g., the small tip of a battery-powered cauterizer; Fine Science Tools, catalog #18010-00) the dura mater is carefully detached from the underside of the bone piece (blunt dissection; Fig. 1*E*,*F*). After exposure, the dura mater is punctured with a small needle or sharp tweezers and gradually opened from the lateral borders of the craniotomy toward the SSS (Fig. 1*G*). Next, the SSS is lifted by the loose dural flaps, and with the help of a ligation aid (Fine Science Tools, catalog #18062-12) a 1.5 absorbable surgical suture (∼10 cm long) is threaded under the sinus (Fig. 1*H–J*). The suture loop is cut and each of the two remaining threads is gently pulled toward the anterior and posterior borders of the craniotomy (Fig. 1*K*,*L*). Here, the SSS is securely ligated and blood flow is interrupted (Fig. 1*M*). Finally, the SSS is cauterized between the ligations and retracted to maximally expose the underlying cortex and the longitudinal fissure (Fig. 1*N*). The simultaneous bilateral targeting of midline structures is now possible.

In humans, occlusions of the SSS cause severe clinical symptoms and are often fatal (Ferro et al., 2004). Yet in rodents, reports suggest that compensatory mechanisms might minimize the potential harmful impact of such obstructions (Stolz et al., 2011; Wang et al., 2019), as long as cortical venal blood flow is preserved, SSS blockage alone does not alter cerebral blood flow, nor does it cause infarction and hemorrhage (Nakase et al., 1996; Röttger et al., 2005). To examine possible adverse side effects of SSS blockage, we compared the results of a battery of motor, memory function, and general behavior tests, in a group of SSS-transected rats (ST group) with those of age-matched controls (sham group), submitted to all surgical procedures except transection. All animals were tested at baseline (presurgery), and at four and eight weeks postsurgery (for details, see Materials and Methods). Furthermore, to evaluate possible mechanical or ischemic damage, we performed a histopathological study of representative brains from animals in each group.

### Transecting the SSS does not impair general behavior

Injuries to the SSS can lead to submeningeal hemorrhages and ischemic lesion to the underlying cortex (Chen et al., 1991; Jussen et al., 2017). To test for possible adverse side effects of the SSS transection on general behavior, and evaluate putative sensorimotor deficits induced by focal lesions, we applied an adapted version of the SHIRPA protocol (Rogers et al., 1997; Hatcher et al., 2001), consisting of an observational screening based on Irwin procedure (Irwin, 1968), to all animals of both groups.

Four weeks after the surgery, there were no qualitative differences between SSS-transected (ST group) and aged-matched sham-operated animals as all parameters scored normal in both groups, indicating that transecting the SSS did not induce behavioral alterations that could be indicative of gross neural damage.

### Transecting the SSS does not impair motor behavior

Motor cortical lesions in humans have devastating effects (Heller et al., 1987; Jørgensen et al., 1995). However, rodents seem to recover remarkably well, with little to no lasting impairments in general motor behavior or in dexterous skill execution (Kawai et al., 2015). After confirming that the general behavioral phenotype of ST and sham animals was identical, we asked whether there were alterations in locomotion or exploratory behavior that could be indicative of injury restricted to adjoining primary and secondary motor areas (Neafsey et al., 1986).

**Figure 2.**
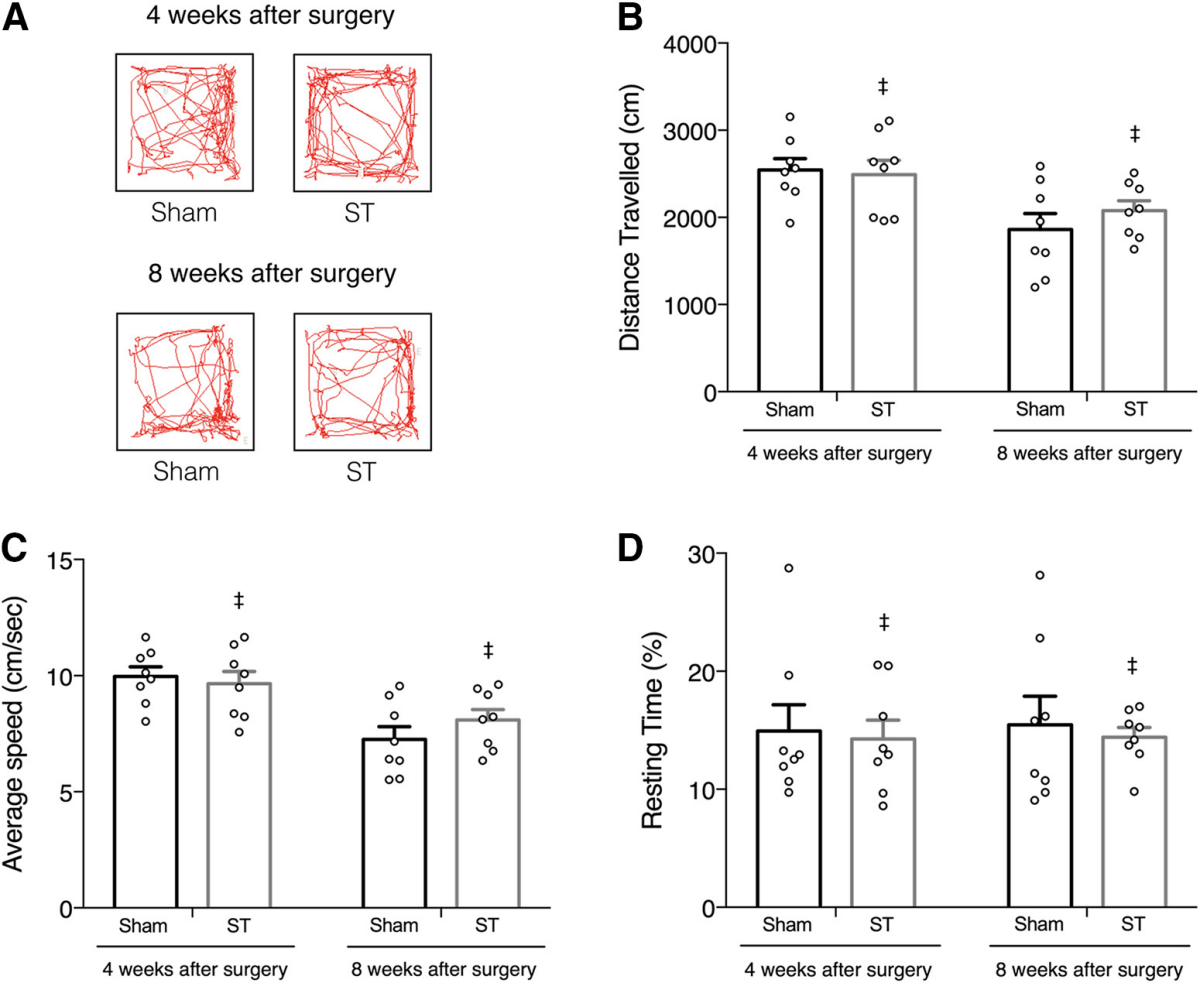
Motor behavior is not impaired by SSS transection. Locomotion and exploratory behavior were assessed by open-field test before surgery (Extended Data Fig. 2-1) and at four and eight weeks postsurgery. ***A***, Representative trackplots of the same ST and sham-operated animal after surgery. No significative differences were observed in total distance traveled (***B***), average speed (***C***), and total resting time (***D***) in all time points tested (*n* = 8, means ± SEM, ‡*p* > 0.05, ST comparing to sham, repeated measures two-way ANOVA followed by Bonferroni’s multiple comparisons *post hoc* test). Permanence in the open-field’s subregions was similar between groups in all time points tested (Extended Data Fig. 2-1).

We recorded exploratory behavior in both groups, during a 5-min open-field test (Fig. 2*A*), and quantified various locomotor parameters. We found that transecting the SSS did not alter the total individually traveled distance (repeated measures two-way ANOVA followed by Bonferroni’s multiple comparisons *post hoc* test, no significant effect of procedure; *F*_(1,14)_ = 0.211, *p* = 0.653; Fig. 2*B*), neither average speed (repeated measures two-way ANOVA followed by Bonferroni’s multiple comparisons *post hoc* test, no significant effect of procedure; *F*_(1,14)_ = 0.199, *p* = 0.662; Fig. 2*C*), nor the total resting time (repeated measures two-way ANOVA followed by Bonferroni’s multiple comparisons *post hoc* test, no significant effect of procedure; *F*_(1,14)_ = 0.189, *p* = 0.670; Fig. 2*D*).

10.1523/ENEURO.0146-21.2021.f2-1 Extended Data Figure 2-1 ST rats exhibit locomotor and exploratory behavior similar to sham-operated animals in all time points tested. Locomotion and exploratory behavior were assessed by the open-field test before surgery and at four and eight weeks postsurgery. ***A***, Representative trackplots of ST and sham-operated animals before surgery. ***B***, No significant differences were found in total distance traveled, average speed and total resting time (*n* = 8, ‡*p* > 0.05, ST compared to sham, two-tailed unpaired *t* test) during presurgery testing. After basal assessment, animals were randomly assigned to experimental (ST) and control (sham) groups. Permanence in the open-field’s subregions was similar between groups in all time points tested (*n* = 8, ‡*p* > 0.05, ST compared to sham, two-way ANOVA followed by Tukey’s multiple comparisons *post hoc* test; ***C***). All values are mean ± SEM. Download Figure 2-1, TIF file.

Furthermore, exploratory behavior was also unaffected by the transection of SSS, as the permanence in the open-field’s subregions was comparable between groups at the time points tested [four weeks after surgery: two-way ANOVA followed by Tukey’s multiple comparisons *post hoc* test, no interaction (*F*_(2,42)_ = 2.002, *p* = 0.148); eight weeks after surgery: two-way ANOVA followed by Tukey’s multiple comparisons *post hoc* test, no interaction (*F*_(2,42)_ = 0.295, *p* = 0.746); Extended Data Fig. 2-1*C*].

In essence, ST rats exhibit normal locomotor and exploratory behavior, comparable to sham-operated animals in all behavioral measures acquired (Fig. 2). These results were stable over time, thus excluding the development of lasting deficits resulting from transecting the SSS.

### Transecting the SSS does not impair short-term spatial memory

**Figure 3.**
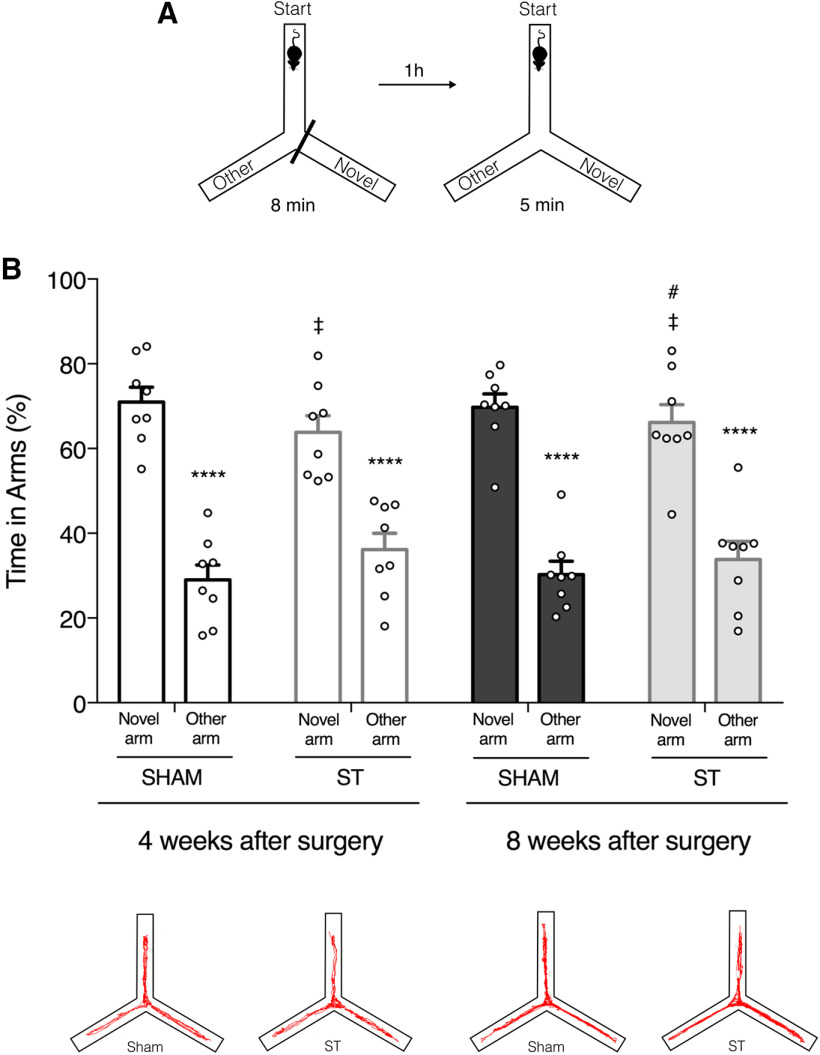
Short-term spatial memory is not affected by SSS transection. Spatial memory performance was assessed by the Y-maze test before surgery (Extended Data Fig. 3-1) and at four and eight weeks postsurgery. ***A***, Schematic representation of the Y-maze test. ***B***, top, Quantification of the time spent by sham and ST animals in novel versus other arm at four and eight weeks postsurgery. Both groups showed preference for the novel arm (*n* = 8, ****p < 0.0001, novel arm compared with other arm, two-way ANOVA followed by Tukey’s multiple comparisons *post hoc* test). The performance of ST and Sham animals was comparable in both time points (*n* = 8, ‡*p* > 0.9999, ST compared with sham, two-way ANOVA followed by Tukey’s multiple comparisons *post hoc* test). No significant differences were found between the performance of ST animals at different time points (*n* = 8, #*p* = 0.9999, time spent in the novel arm at four weeks postsurgery compared with eight weeks postsurgery, two-way ANOVA followed by Tukey’s multiple comparisons *post hoc* test). All values are mean ± SEM (***B***, bottom). The number of transitions between arms was not different between groups at each time point (Extended Data Fig. 3-1). Representative trackplots of the same ST and sham-operated animal at four and eight weeks postsurgery. Within subject analysis showed no significant differences in the performance across time points (Extended Data Fig. 3-1).

Having found that locomotor function was fundamentally preserved, we asked whether memory-dependent behaviors might have been affected by transecting the SSS. Underlying the SSS is the medial mesocortex (anterior cingulate, midcingulate, and retrosplenial), which communicates bi-directionally with the hippocampus and is proposed to be critical for spatial memory and memory-guided behaviors (Ferreira-Fernandes et al., 2019). Considering the proximity of the transected SSS to the anterior cingulate cortex, we assessed the consequences of possible damage to these structures using the Y-maze test for short-term spatial reference memory (Fig. 3*A*).

10.1523/ENEURO.0146-21.2021.f3-1 Extended Data Figure 3-1 Short-term spatial memory performance of ST rats is similar to sham-operated animals in all time points tested. Spatial memory performance was assessed by the Y-maze test before surgery and at four and eight weeks postsurgery. ***A***, Representative trackplots of ST and sham-operated animals before surgery. ***B***, Quantification of the time spent by sham and ST animals in novel versus other arm at baseline assessment. Both groups showed preference for the novel arm (*n* = 8, means ± SEM, ****p < 0.0001, novel arm compared to other arm, two-way ANOVA followed by Tukey’s multiple comparisons *post hoc* test). After basal assessment, animals were randomly assigned to experimental (ST) and control (sham) groups. ***C***, Quantification of the number of transitions between arms. No differences were found between groups (*n* = 8, means ± SEM, ‡*p* > 0.05, ST compared to sham, repeated measures two-way ANOVA followed by Bonferroni’s multiple comparisons *post hoc* test). ***D***, Quantification of the time spent by the same animal in the novel arm at different assessments. Download Figure 3-1, TIF file.

ST animals showed a preference for the novel arm similar to sham-operated animals at four and eight weeks after surgery (two-way ANOVA followed by Tukey’s multiple comparisons *post hoc* test, significant novelty effect; *F*_(1,56)_ = 179.9, *p* < 0.0001; Fig. 3*B*).

No significant differences were found between the performances of ST and Sham animals after the procedure (Fig. 3). Two-way ANOVA followed by Tukey’s multiple comparisons *post hoc* test indicates no significant effect of SSS transection (*F*_(3,56)_ = 0, *p* > 0.9999) nor interaction (*F*_(3,56)_ = 1.534, *p* = 0.2158).

Furthermore, within subject analysis showed no significant differences in the performance levels of ST animals across time points (repeated measures one-way ANOVA, *F*_(1.517,10.62)_ = 0,192, *p* = 0.769; Extended Data Fig. 3-1*D*).

To exclude possible effects of locomotor activity on exploratory behavior, confounding the latter, we counted and used the number of transitions between arms as an indirect measure of general locomotor activity. Once again, no differences were found between groups at each time point [repeated measures two-way ANOVA followed by Bonferroni’s multiple comparisons *post hoc* test, no significant effect of procedure (*F*_(1,14)_ = 1.202, *p* = 0.291) nor interaction (*F*_(2,28)_ = 1.819, *p* = 0.181); Extended Data Fig. 3-1*C*].

Overall, transection of the SSS had no transient or long-lasting impact on short-term spatial reference memory.

### Brain tissue viability is preserved after SSS transection

After confirming that general behavior, motor, and memory functions were fundamentally preserved, we performed histopathological analysis of brain samples to test tissue integrity and viability.

**Figure 4.**
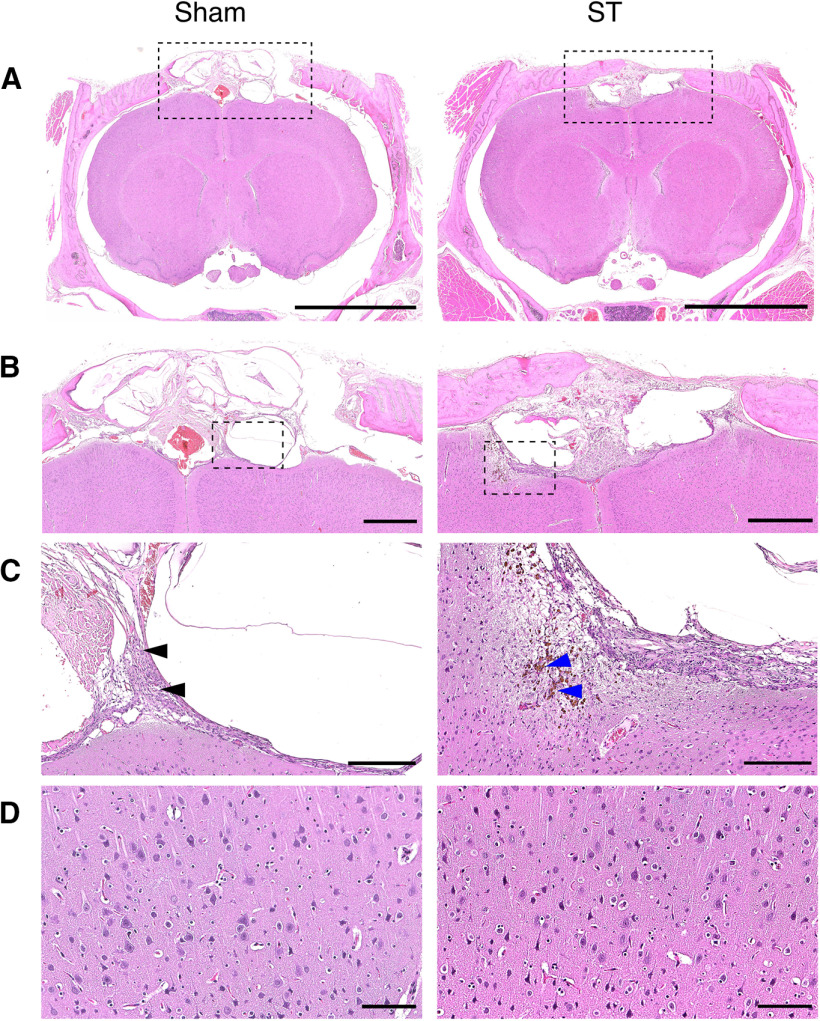
Transection of SSS does not affect brain tissue viability. Histopathological analysis indicates no extensive neural damage or focal lesion 10 weeks after SSS transection. ST animals are comparable to sham-operated animals in all features analyzed. ***A***, Low magnification of brain, skull and meninges stained with hematoxylin and eosin. Scale bar: 5000 μm. ***B***, Brain and skull magnification. Features of normal surgical wound healing were found within the skull and meninges in the craniotomy area of both groups. Note the scar tissue (scored as moderate). No alterations were found in the cerebral parenchyma. Scale bar: 1000 μm. ***C***, Magnification of scar tissue (scored as moderate), composed mainly of fibroblasts, neovascularization, and macrophages (black arrows) with occasional hemosiderin accumulation (blue arrows). Scale bar: 250 μm. ***D***, High magnification of cerebral parenchyma. No alterations were observed in the neurons and glial cells. Scale bar: 100 μm. Histologic damage is not correlated with behavioral performance and did not involve ventricular alterations (Extended Data Fig. 4-1).

A total of 10 weeks after surgery, the skull and meninges in the craniotomy area showed typical features of normal wound healing in both groups (Fig. 4). There was moderate scar tissue formed in the bone and overlying skin, mainly composed of fibroblasts, neovascularization, and macrophages, with occasional hemosiderin accumulation (Fig. 4*B*,*C*). In a few cases, this extended through the superficial brain parenchyma in both ST and sham-operated animals.

10.1523/ENEURO.0146-21.2021.f4-1 Extended Data Figure 4-1 Histological damage is not correlated with performance and did not involve ventricular alterations. ***A***, The extent of damage was quantified in three animals from each group, by measuring the lesioned area in five serial sections per animal (across 1.5 mm) and normalizing to the whole-brain area. No correlation was found between histological damage and Y-maze performance at eight weeks postsurgery (sham: *r*^2^ = 0.19, *p* = 0.71; ST: *r*^2^ = 0.87, *p* = 0.23; Pearson’s correlation). No significant differences were found in histological damage between groups (*n* = 3, *p* > 0.05, ST compared to sham, two-tailed unpaired *t* test). All values are mean ± SEM. ***B***, Normodimensioned lateral ventricles of sham and ST animals. Scale bar: 500 μm. Download Figure 4-1, TIF file.

Histologic damage was assessed in three animals from each group by measuring the lesion area in five serial sections per animal (across 1.5 mm), centered in the zone where the transection was performed, and normalizing it to the whole-brain area. Percentage damage was lower than 0.5% in both groups and not significantly different (sham 0.245 ± 0.144, *n* = 3; ST 0.249 ± 0.119, *n* = 3, two-sided unpaired *t* test, *t*_(4)_ = 0.0206, *p* = 0.985). Furthermore, no correlation was found between histologic damage and behavioral performance (*p* > 0.05; Extended Data Fig. 4-1*A*).

Since any surgery involving dura mater opening can potentially lead to CSF leakage (Kawai et al., 2014), we inspected ventricular dimensions and confirmed the absence of sizable alterations (Extended Data Fig. 4-1*B*). At the cellular level, no major lesions were found in the gray and white matter, and no alterations were observed in neurons or glial cells (Fig. 4*D*).

Histopathological analysis denotes no relevant alterations in ST animals that could be indicative of significant neural damage, or focal lesions. Therefore, SSS transection procedure does not affect viability and integrity of underlying brain tissue.

### SSS transection enables microinjections and simultaneous tetrode recordings in deep hypothalamic nuclei

Having established that ST animals have normal motor and memory functions, and that tissue health is preserved, we next validated our method by injecting rats with viral vectors and neural tracers, and implanting octrode drives in deep hypothalamic areas. We used octrodes because of their increased rigidity. However, only four channels (i.e., tetrodes) per octrode were effectively used for recordings.

**Figure 5.**
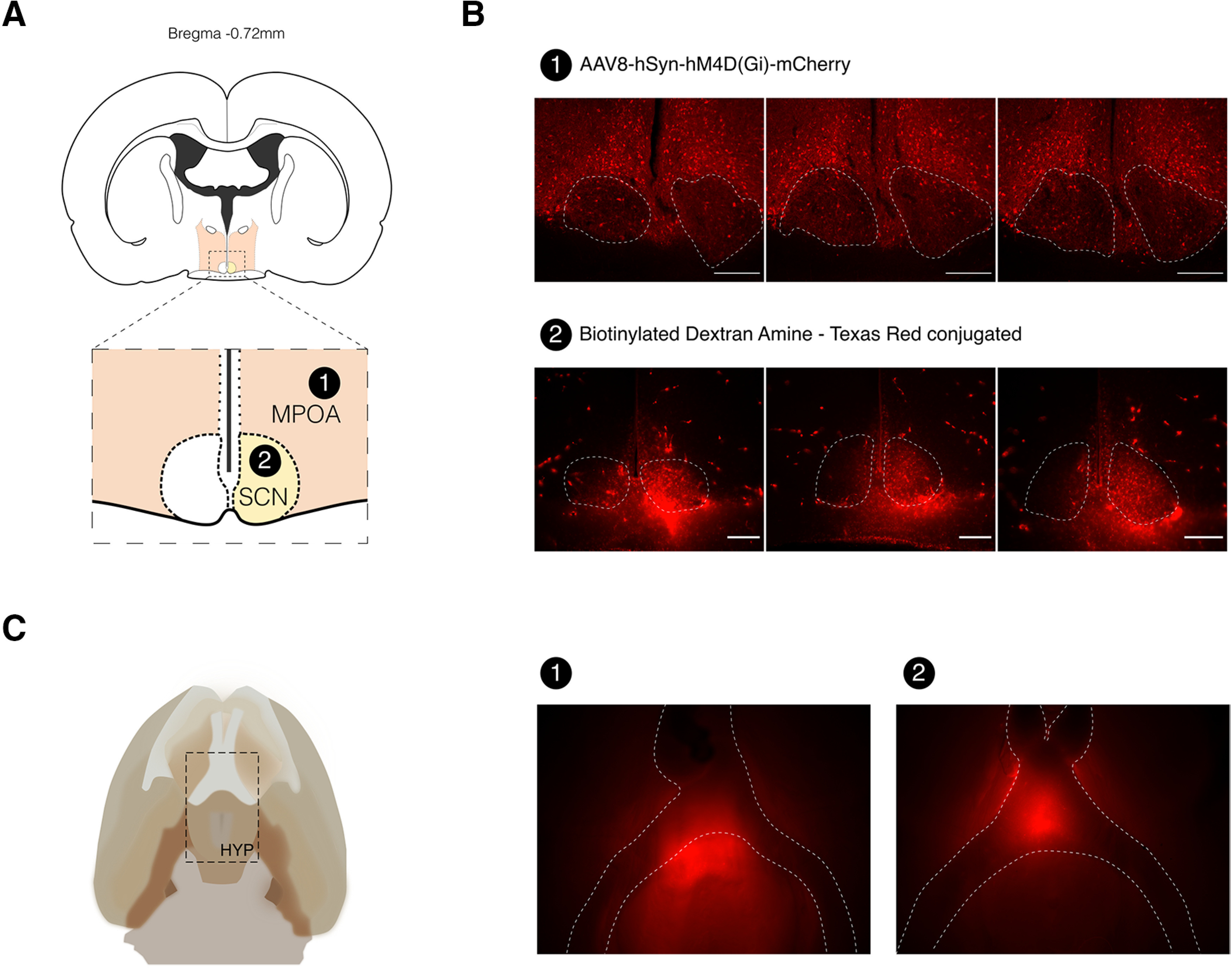
SSS transection allows accurate microinjection of deep hypothalamic nuclei. ***A***, Schematic representation of the target areas used for microinjection: (1) MPOA and (2) SCN. ***B***, Photomicrograph of coronal sections showing extensive AAV8-hSyn-hM4D(Gi)-mCherry expression in the MPOA at five weeks postinfection (1) and strong BDA–Texas Red uptake in the SCN at 11 d postinjection (2). Scale bars: 250 μm. ***C***, Schematic drawing of the ventral surface of the rat brain detailing hypothalamus’ location (left). Whole-brain imaging confirms the presence of fluorescence in deep hypothalamic structures surrounding the optical chiasm, five weeks after injection in MPOA (1) and 11 d postinjection in SCN (2; right). All injections performed using the same virus and tracer showed similar patterns (Extended Data Fig. 5-1).

After transecting the SSS, animals were stereotaxically injected with either a viral vector (AAV8-hSyn-hM4D(Gi)-mCherry, *n* = 2) in the MPOA or an anterograde neural tracer (BDA–Texas Red labeled, *n* = 7) in the SCN (Fig. 5*A*).

10.1523/ENEURO.0146-21.2021.f5-1 Extended Data Figure 5-1 SSS transection allows consistent microinjection of deep hypothalamic nuclei. Photomicrograph of coronal sections showing extensive AAV8-hSyn-hM4D(Gi)-mCherry expression in the MPOA at five weeks postinfection (1) and strong BDA–Texas Red uptake in the SCN at 11 d postinjection (2). SSS allows consistent targeting of MPOA and SCN across subjects. Scale bar: 250 μm. Download Figure 5-1, TIF file.

Success ratio was defined as the ability to vertically reach our target area without extensive bleeding, damage to underlying brain tissue or causing animal death. Although precise targeting of deep hypothalamic nuclei is dependent on several other variables (i.e., correct determination of coordinates, appropriate head leveling and fixation), this method particularly improves the accuracy of the injections in deep structures by allowing correct determination of the dorsoventral coordinate “zero,” on the brain surface (DV = 0).

Accordingly, five weeks (viral vector) or 11 d (BDA) postinjection, fluorescence microscopy imaging shows extensive viral expression or tracer uptake in the targeted regions (Fig. 5*B*) as well as anterograde spreading to hypothalamic nuclei (Fig. 5*C*) in all the nine ST and injected animals. In three animals, the needle tract is visible within the nucleus (Extended Data Fig. 5-1*B*, #1, #3, #4). The remaining animals have staining within the nucleus consistent with correct targeting, indicating accurate stereotaxic delivery of compounds in the MPOA and SCN. All injections performed using the same virus and tracer showed similar patterns (Extended Data Fig. 5-1).

As transecting the SSS allowed accurate targeting of deep hypothalamic nuclei with minimal increase in surgery time or surgical complexity, we then tested our method for the recording of neural activity in deep midline brain areas.

After SSS transection, we implanted two animals with octrodes. The first animal served as a targeting test and was implanted with a bundle of octrodes dipped in DiI stain not connected to any recording system. One day after the surgery, electrolytic lesions were performed under anesthesia and the animal was killed. Postmortem analysis revealed accurate bilateral targeting of SCN (Extended Data Fig. 6-1*A*).

**Figure 6.**
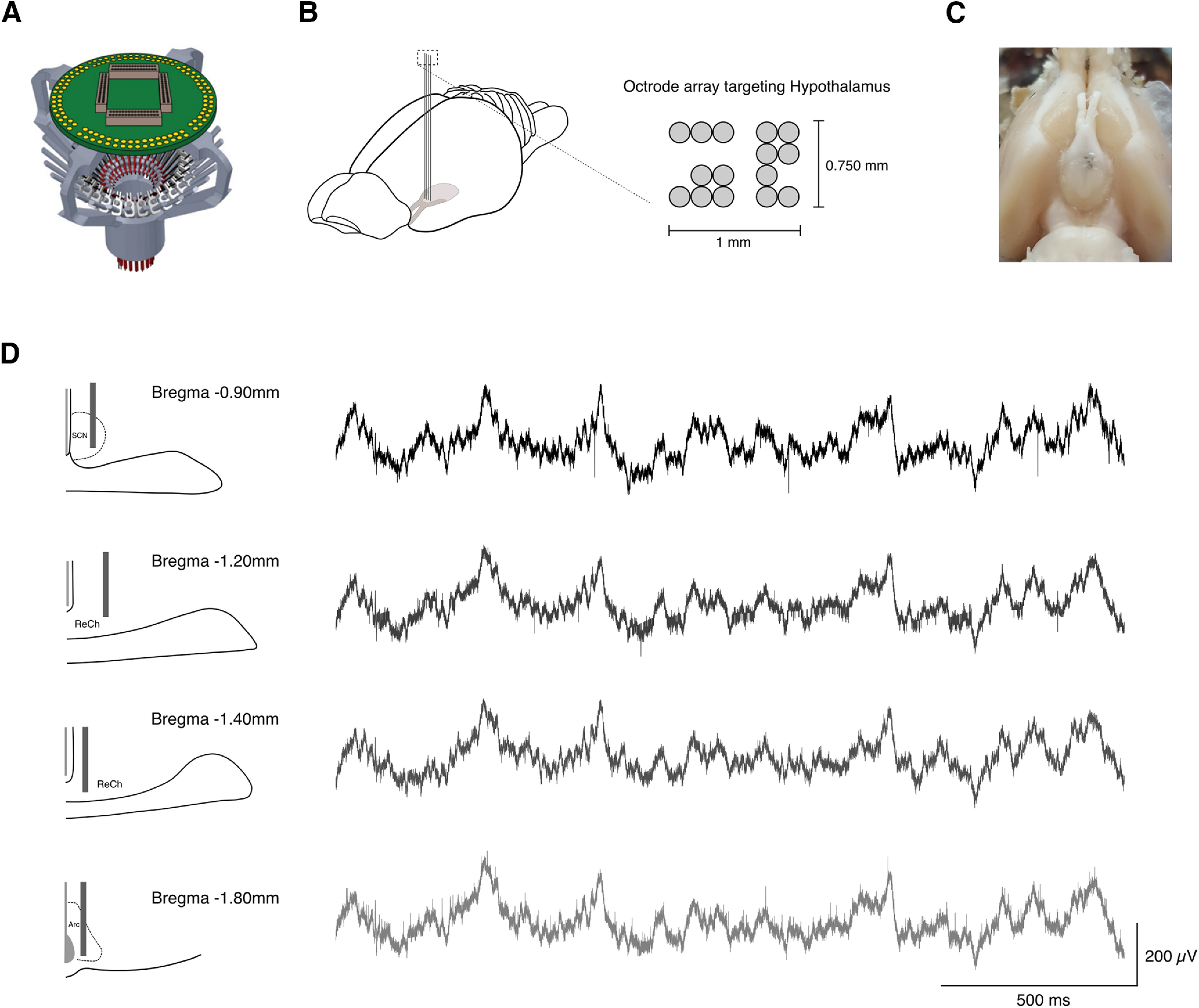
SSS Transection enables multisite recording of deep midline brain areas. ***A***, Representation of the SLIQ hyperdrive used to chronically record neural activity across multiple regions in freely moving animals. ***B***, Schematic representation of the experimental approach (left). An array of 15 octrodes was chronically implanted to simultaneously record neural activity from different hypothalamic nuclei (right). ***C***, Photograph of ventral brain surface, confirming octrode targeting of deep hypothalamic areas surrounding optical chiasm. ***D***, Schematic of tetrode recording of neural activity from SCN, ReCh, and Arc (left). Two seconds of example LFP recorded simultaneously from SCN, ReCh, and Arc (right). Anatomical location of octrode tips was confirmed by histologic analysis (Extended Data Fig. 6-1*B*).

The second animal was chronically implanted with a functional hyperdrive (Liang et al., 2017; Fig. 6*A*) carrying 15 independent octrodes (Fig. 6*B*) to simultaneously record neural activity (Local Field Potentials (LFP) and single units) from anterior and intermediate hypothalamic nuclei in a freely moving animal. Octrodes were manually lowered to the desired depth and glued in place, remaining fixed throughout the recording experiment.

10.1523/ENEURO.0146-21.2021.f6-1 Extended Data Figure 6-1 SSS transection allows multisite recording from deep midline brain structures. ***A***, Postmortem analysis revealed accurate bilateral targeting of SCN in preliminary targeting test, as indicated by the electrolytic lesion and DiI staining. Cell nuclei are stained with Hoechst in blue fluorescence. Octrode tips were dipped in DiI stain (red fluorescence). Scale bar: 250 μm. ***B***, Histological verification of octrode tracks confirms targeting of SCN, ReCh, and Arc. Cell nuclei are stained with Hoechst in blue fluorescence. White arrows indicate octrode tips. Scale bar: 0.50 cm. Download Figure 6-1, TIF file.

Using this method, we were able to successfully perform simultaneous single unit and LFP recordings from SCN, retrochiasmatic area (ReCh), and arcuate nucleus (Arc; Fig. 6*D*) as confirmed by histologic verification of octrode tracks (Fig. 6*C*; Extended Data Fig. 6-1*B*). Although aiming at simultaneous bilateral recordings, postmortem histologic verification revealed unilateral targeting and final coordinates more posterior than expected, most likely because of unleveled drive and octrode insertion during the surgery.

**Figure 7.**
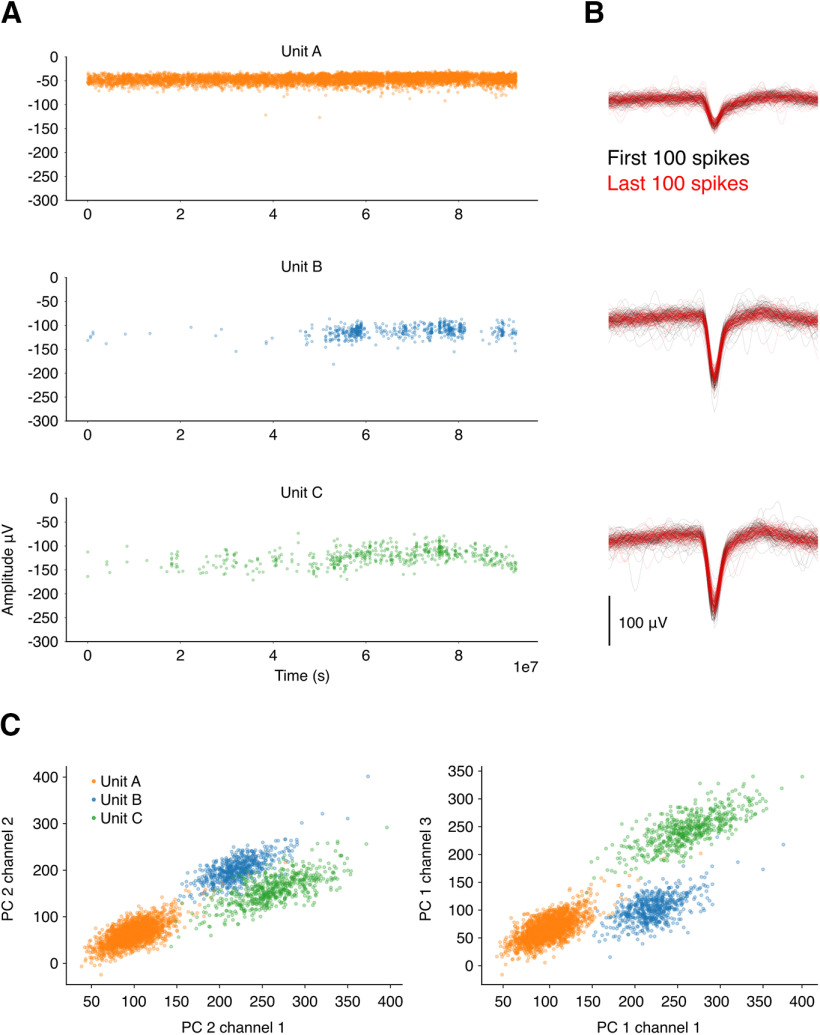
Single units are well isolated and stable over the recording period. ***A***, Amplitude over the duration of an example recording session for three units simultaneously recorded from the same octrode. This recording was performed inside a sleep-box, where the animal was free to move. Units B and C increase their firing rates at a later moment of the recording session when the animal starts to sleep or stays immobile for long periods of time. ***B***, First and last 100 spikes for each unit recorded from the channel where the highest amplitude was detected. Note the significant overlap between early and late waveforms. ***C***, PC scores for the same units as above.

Units were well isolated (Fig. 7*C*) and the recordings were stable across the duration of a session (Fig. 7*A*,*B*). Units B and C started firing when the animal started sleeping or stopped moving inside the sleep-box (Fig. 7*A*). The number of units varied between recording days. Eight days after the surgery the number of units was relatively low and increased in the second recording session. From this point onwards, we observed a continuous decline in the number of units, as expected in chronic targeting with fixed probes.

Overall, our results show that transecting the SSS is a viable and safe method to gain direct, vertical access to deep brain structures, as well as superficial ones underlying the SSS. Rats with the transected sinus (ST group) show no behavioral alterations and tissue integrity is minimally disrupted, allowing for accurate recording of neural activity without deleterious effects of extensive brain damage or postsurgical hemorrhage.

## Discussion

We developed a surgical method that enables direct access to brain regions underlying the SSS. Key steps to this strategy are the ligation and transection of the SSS, enabling retraction of this major venous vessel, exposing the sagittal fissure. We found that, in animals with transected SSSs, the interruption of normal blood flow and surgery-associated mechanical damage to cortical structures in the vicinity of the transected SSS, had no measurable negative impacts. We performed a comprehensive battery of postsurgical testing and found no evidence of compromises to general behavior, short-term memory or motor function. Moreover, histologic examination confirmed that SSS transection did not increase brain damage or inflammation when compared with sham operated animals, indicating preserved tissue viability. Our data show that transected animals are comparable to sham animals in all parameters measured. This might be surprising since the SSS is a vessel of large dimensions, and in fact, in humans, its interruption can have devastating consequences (Ferro et al., 2004). In rodents, it has been suggested that consequences of occlusions to the SSS can be minimized by compensatory mechanisms (Stolz et al., 2011; Wang et al., 2019). SSS blockage alone does not alter cerebral blood flow, nor does it cause infarction and hemorrhage (Nakase et al., 1996; Röttger et al., 2005), when care is taken to minimally impact cortical venal blood flow, as is the case in our methodology. Importantly, this evidence suggests that particular care needs to be taken to preserve the surrounding vasculature to prevent neural damage. Although we cannot rule out acute effects of SSS transection on brain function, all behavior measures, including the SHIRPA (performed at four weeks), which better examines possible neurologic deficits, did not suggest the presence of significant neural damage. Moreover, at 10 weeks after surgery we can only detect typical features of normal wound healing in both groups, with preserved cortical morphology and no alterations in neurons or glial cells. Therefore, it seems unlikely that relevant acute damage, beyond the normal postsurgical condition, is impacting function later.

After establishing the feasibility of this method to expose the sagittal fissure and confirming that animal health and behavior were preserved, we tested its usefulness in two different neural targeting problems. We started by injecting deep midline hypothalamic nuclei with different neural tracers in a group of animals. Our results showed accurate delivery of tracers in the targeted areas with a minimal time cost for resecting the SSS (∼2 h from anesthesia to wound closure) and 100% success ratio. Next, we tested its advantages in a particularly challenging implantation surgery: hyperdrive (Liang et al., 2017) targeting deep midline hypothalamic nuclei. Successful implantation allowed us to record single unit activity and LFP simultaneously across target regions, extending the usefulness of our method to complex implantation surgeries targeting midline structures.

Given adequate training and surgical care, we found the procedure to add few risks to surgeries, as demonstrated by the extremely low mortality rate. From a total of 28 rats only one animal died during surgery and none during the recovery period. This demonstrates the feasibility of this approach especially in implantation techniques for *in vivo* recordings and manipulations of neural activity in freely behaving animals, in which survival is crucial. One factor directly influencing surgical success is the existence of interindividual differences in brain vasculature. Large bridging or cortical veins located in the transected region of the SSS might warrant cancelling the procedure if slight readjustment of the target coordinates is not feasible. The use of either very young or old animals might also constitute possible complications. Particularly in older animals, adhesion of the dura mater to the skull tends to be higher (personal observations), making the extraction of the bone flap to increase the risk of rupturing the SSS in this initial step. However, we believe that with adequate testing and optimization, this method can be adapted to animals of different ages. Also, since our surgical site was relatively anterior and we did not test the procedure at more posterior coordinates where the SSS is thicker, we cannot claim feasibility across the whole SSS extension. Nonetheless, at least one study employing a surgical procedure to expose the pineal gland, located under the confluence of the SSS and the transverse sinuses, reported no surgical complications and a comparably high success rate in the removal of a more posterior section of SSS (Maganhin et al., 2009). This strongly suggests that the procedure might be applicable in other sections of the SSS.

Since there is preservation of the dorsal venous system (SSS, transverse sinuses, rostral and caudal rhinal veins) between rats and mice (Xiong et al., 2017) it seems feasible that this method should be applicable to mice, with minimal adaptations. This method seems to be particularly useful for circumstances requiring wide midline unimpeded access. Implanting larger footprint implants at such coordinates requires SSS to be chronically kept away from the midline after implantation (Wirtshafter et al., 1979). Additionally, most drive designs preclude assembling bundles with lateral-to-midline converging angles for bilateral targeting. In these circumstances, dislocating the SSS or attempting angled approaches might not be viable options, could cause extensive bleeding or reduce targeting accuracy. Our method circumvents these difficulties, while simplifying targeting and increasing success rates. We believe that the added benefit of transecting the SSS largely outweighs the increased surgical complexity and duration (∼38 min).

Using the procedures we now report, we have demonstrated precise delivery of neural tracers, as well as chronic recordings of neural activity in the midline hypothalamic nuclei during unrestrained behavior. Bilateral access to deep brain regions, particularly using highly sensitive devices/probes with a comparably large footprint, was previously not possible. At a minimal time cost, this method provides direct access for manipulation and recording of activity in brain areas previously obstructed by the SSS.

## Synthesis

Reviewing Editor: Liset Menendez de la Prida, Instituto Cajal CSIC Decisions are customarily a result of the Reviewing Editor and the peer reviewers coming together and discussing their recommendations until a consensus is reached. When revisions are invited, a fact-based synthesis statement explaining their decision and outlining what is needed to prepare a revision will be listed below. The following reviewer(s) agreed to reveal their identity: Nelson Totah.

Your ms was independently evaluated by one reviewer and myself. Then we discussed our views and reached consensus. We both agree this is a very interesting paper describing a new surgical method to solve accesability issues to midline brain structures in rats due to the location of the superior sagittal sinus (SSS). We value the detailed description and assessment of potential effect of SSS transection and retraction at the anatomical and functional levels. We also value your proof-of-principle data on the validity of the approach when applied to neural tracing experiments and tetrode recordings.

The approach includes transection and retraction a portion of the SSS. We have concerns regarding the risk of complications causing local brain isquemia and other injuries. While the report addresses this by evaluating a number of anatomical and functional variables, you may need to consider the following issues.

1- Most of the anatomo-pathological data provided is 10 weeks after SSS transection, and compensatory mechanisms may occlude acute effects running at the time of some experiments (4 weeks). We would like you to address this either by providing some data at 4 weeks after surgery, or by discussing this critique in discussion.

2- To reinforce the absence of effect it may be good to evaluate whether variability of behavioral performance can be explained by some anatomical variable. While performance of sham and SSS groups look quite overlapping, a correlation analysis, even if in a subset of animals may be useful. This can also help to provide some quantification of histopathological analysis (Fig.4), even if semi-quantitatively. Actually, you mention damage is limited to less than 5% of the brain mass (line 396) but it is unclear how did you test it. Similarly, it is mentioned (line 398) that “... transection of SS did not affect intracranial pressure..” as judged by choroid plexus and ventricle dimensions, but data is not provided.

3- Data from tracing experiments and tetrode recordings is not quantified. For tracing experiments, it may be good to add some quantitative assessment of the success ratio at least for the n=7 group. For tetrode recordings, we have concerns this is data from a single animal. While it may account as proof-of-principle, it may be good to provide some additional evidence, or at least to improve reporting in terms of recording quality (e.g. unit isolation versus MUA, recording stability, etc..). Actually, while method describes some spectral analysis of ephys traces there is nothing shown in results.

Other comments:

4- In reading a bit about the background of this paper, we came across this study (https://www.pnas.org/content/111/52/18739; Low, Gu, and Tank 2014 PNAS). We feel it is useful to show how others have addressed the challenge of this sinus physically impeding implantation of midline structures. We encourage you to cite this paper together with any other, to illustrate how various approaches have beed used and to discuss how yours may benefit the implantation of large lenses for imaging medial structures.

5- You claim this is a general procedure for rodents, but we wonder whether the SSS morphology is similar in rats and mice? Given the interest of mice for the community we feel it maybe useful to know more about the vascular morphology of the SSS and associated branches in both species. For instance, is there an inferior sagittal sinus?

6- Resection involves only the anterior part of the SSS. Intervention at more posterior parts may lead to different complications (the sinus is thicker there). Maybe useful to comment

7- Line 86: “..to partially transect..” why partial? The transection procedure looks like it compromises the blood flow. Please, clarify

8- In the methods, the stereotaxic coordinates for the tetrode implants were not provided. Also, there is no description of the hyperdrive and tetrode dimensions. For instance, what is the distance between the different tetrodes and the footprint dimension? Are recordings bilateral or unilateral? Please, provide some additional details.

9- Please, state whether or not the SHIRPA assessments were blinded

10- page 13, line 363, we suggest using the word “similar” instead of “comparable” because one of us misread the sentence twice and thought it said that the ST rats shows a preference for the novel arm “compared to” the sham rats.

11- In figure 1F, it would be helpful adding something like a dotted line and errors showing the location and direction of the dural incisions. This visual aid would complement the text and help with understanding how to replicate the method.

12- Was the anesthesia isofluorane and also Ketamine/Medetomidine? Or was isofluorane used to slightly sedate the animals before injecting the Ket/Med?

13- Line 118: “.. no animal was lost during surgery” but then in discussion line 490: “From a total of 26 frats only one animal died during surgery...”. Please check and clarify.

14- Line 135: ...after a 5 minutes rest period” What does it mean? You mean 5 min of no injection?

15- Methods: please clarify post-surgical treatment. Are analgesics or anti-immflamatory treatment used? Any antibiotics? For how long?

16- Methods of pathological analysis: is the entire head cut in 4um sections. How is the decalcified procedure? Please, specify these procedures with sufficient details.

17- Regarding tetrode recordings: how clear is penetration and retraction in terms of bleeding or any other damage? This may compromise microdrive movements and/oor probe recovery after experiment. Giving this is a major motivation for adopting this surgical procedure it may be useful to know whether it solves this issue.

18- Line 342: “... all timepoint tested” Only 4 and 8 week time points are tested. Check line 364 also.

19- Lines 418 and 429: it should refer to Fig.5 and 6 not 4 and 5.

20- Lines 508: access with an angle make the approach possible, even if not optimal, as described in the introduction.

## Author Response

Dear Dr Liset Menendez de La Prida,

Thank you for your message, and for the valuable feedback we received concerning our

manuscript. Please extend our thanks to the reviewers for their constructive criticisms and

comments, prompting additional analyses, discussions, and clarifications. These have

significantly improved the quality of the manuscript. We are hereby submitting a new

version of our manuscript, including revisions and additional data.

In this revised version, we clarified the advantages of our surgical method relative to

other approaches that have been used to target brain structures, and discussed its

applicability in other species, and in different brain targets.

We have also quantified the histological damage, and explored a potential correlation

with behavioral performance, to meet the concerns expressed by Rev 1 regarding

histopathological consequences of our method.

Lastly, we quantified the results of neural tracing experiments and of tetrode-based

neural recordings, thus providing a quantitative assessment of the success ratio obtained

after targeting deep hypothalamic nuclei for neural tracer injection, and neural recordings,

in our “proof-of-principle” implanted animal. These additional analyses resulted in a new

figure added to the manuscript, reporting stability, isolation, and number of recorded units

throughout the experiment.

A point-by-point reply to the Reviewers’ concerns is attached, as well as a visual

abstract of our manuscript. To provide more information on the surgical procedure, we

have uploaded a step-by-step protocol to our institute’s server and a permanent URL was

generated by IT department. The URL was provided only in the Clean Copy of the article

to comply with blinded review. A copy of the detailed protocol is attached.

We hope that all the concerns have been addressed, and that, with the data and

corrections included in this revised version, you will find our manuscript worthy of

publication in eNEURO.2

Major points:

1) The approach includes transection and retraction a portion of the SSS. We have

concerns regarding the risk of complications causing local brain isquemia and other

injuries. While the report addresses this by evaluating a number of anatomical and

functional variables, you may need to consider the following issues. Most of the anatomopathological data provided is 10 weeks after SSS transection, and compensatory

mechanisms may occlude acute effects running at the time of some experiments (4

weeks). We would like you to address this either by providing some data at 4 weeks after

surgery, or by discussing this critique in discussion.

We acknowledge that deleterious acute effects to the tissue cannot be excluded as we

did not perform histological tests at earlier time points. However, all behavior measures,

including the SHIRPA (performed at 4 weeks), which better examines possible

neurological deficits, did not suggest the presence of relevant neural damage.

Although we cannot exclude acute effects, we have now calculated the percentage of

lesioned relative to whole brain to be lower than 0.5% at 10 weeks after the surgery. There

were no differences between groups, neither did the extent of lesioned tissue correlate

with behavioral performance (see point 2). Moreover, the observed histological alterations

are typical features of normal wound healing, and present in both groups, with preserved

cortical morphology, and no loss of neurons or glial cells. It is unlikely that relevant acute

damage would result in full healing of nervous tissue to the point of it looking normal by

week 10.

It is likely that sealing the craniotomy with agarose minimized early tissue instability

(see also point 2C), thus contributing for healing to occur with minimal damage.

We have added a paragraph in the discussion to address this point (line 544 onwards):

"Although we cannot rule out acute effects of SSS transection on brain function, all

behavior measures, including the SHIRPA (performed at 4 weeks), which better examines

possible neurological deficits, did not suggest the presence of significant neural damage.

Moreover, at 10 weeks after surgery we can only detect typical features of normal wound

healing in both groups, with preserved cortical morphology and no alterations in neurons

or glial cells. Therefore, it seems unlikely that relevant acute damage, beyond the normal

post-surgical condition, is impacting function later.”3

2)

A) To reinforce the absence of effect it may be good to evaluate whether variability of

behavioral performance can be explained by some anatomical variable. While

performance of sham and SSS groups look quite overlapping, a correlation analysis, even

if in a subset of animals may be useful. This can also help to provide some quantification

of histopathological analysis (Fig.4), even if semi-quantitatively.

To address whether histological damage was correlated to behavioral performance, we

quantified lesion extent and plotted it against performance in the Y-Maze at 8 weeks postsurgery, for 3 animals in each group (see graph below). No correlation was found between

the two variables in both groups (Sham: r2

= 0.19, p = 0.71; ST: r2

= 0.87, p = 0.23),

indicating no effect of lesion on behavioral performance. Also, the differences between the

slopes are not significant (p = 0.1379).

We have now included a reference to this in the results section (line 439 onwards):

"Furthermore, no correlation was found between histological damage and behavioral

performance (p > 0.05) (Figure 4-1A).”

B) Actually, you mention damage is limited to less than 5% of the brain mass (line 396) but

it is unclear how did you test it.

We have now quantified the extent of damage in 3 animals from each group, by

measuring the lesioned area in five serial sections per animal (across 1.5 mm) - centered

in the zone where the transection was performed - and normalizing this to the whole brain

area.

Percentage damage was lower than 0.5% in both groups and not significantly different

(Sham 0.245 {plus minus} 0.144, n=3; ST 0.249 {plus minus} 0.119, n=3, two-sided unpaired t-test, t(4) = 0,0206,

p = 0.985).

This quantification was added to the manuscript (line 434 onwards).4

C) Similarly, it is mentioned (line 398) that “... transection of SS did not affect intracranial

pressure..” as judged by choroid plexus and ventricle dimensions, but data is not provided.

Intracranial pressure (ICP) is determined by the three components occupying the

intracranial space: the cerebrospinal fluid (CSF), the blood and the brain (Kim et al.,

Neurotrauma, 2012). Disruptions in the CSF secretion or drainage will have an impact on

this system’s homeostasis, leading to ICP disturbance (Leinonen et al., Handbook of

clinical neurology, 2018). As previously described (Kawai et al., Neurol Med Chir, 2014),

neurosurgical procedures involving extensive durotomy cause transdural CSF leakage,

creating an imbalance to the CSF circulatory dynamics. In our first attempts of SSS Transection, the craniotomy (4x4mm) was performed without sealing the durotomy at the

end of the surgical procedure. Increased ventricular size resulting in compression of

adjacent structures was observed in these animals, when sacrificed 5 weeks after the

surgery, suggesting an imbalance in CSF homeostasis (see figure below).

We thus adapted the surgical protocol to include sealing the durotomy with agarose,

which completely prevented CSF homeostasis disturbance, and became our standard

procedure, as described in the detailed protocol (attached to this letter). In spite of this,

and since any surgery involving severing the dura can potentially lead to CSF leakage, we

inspected the ventricular dimensions to look for sizable alterations (Figure 4-1B) in

subsequent animals, confirming its absence.

We have revised the text to state exactly what was done (line 442):

"Since any surgery involving dura mater opening can potentially lead to CSF leakage

(Kawai et al., 2014), we inspected ventricular dimensions and confirmed the absence of

sizable alterations (Figure 4-1B).”5

3) Data from tracing experiments and tetrode recordings is not quantified

A) For tracing experiments, it may be good to add some quantitative assessment of the

success ratio at least for the n=7 group.

We defined success ratio as the ability to vertically reach our target area without

extensive bleeding, damage to underlying structures, or animal death - frequent practical

difficulties without SSS-transection.

Although the ability to accurately target these deep hypothalamic nuclei is dependent on

several other variables (i.e. correct determination of AP-ML coordinates, appropriate head

leveling and fixation), SSS-transection effectively allows for increased accuracy in

determining the DV “zero” coordinate (DV=0), since the SSS no longer occludes the brain

surface. As such, the procedure allows for improved accuracy in targeting midline brain

regions, a main motivation for the development of this method.

In the experiment that motivated this methodology (targeting the SCN), 100% of

injections mistargeted the nucleus in all (5/5) animals that survived the injection surgery

without SSS-transection (data not shown). The injection was either too dorsal or ventral

than intended (namely perforating the optic chiasm) indicating inaccurate DV=0

determination, even after lateral mobilization of SSS. To overcome this, we performed the

first attempts of SSS-transection in 2 animals, obtaining correct injection targeting.

In the experiment we now report, 100% of the targeting attempts (9 animals) after SSS

transection resulted in accurate neural tracer injection (Figure 5-1), 3/9 had, in addition to

this, a visible needle track within the nuclei (Figure 5-1B #1, #3, #4).

The above details were added to the results section (line 465).

B) For tetrode recordings, we have concerns this is data from a single animal. While it may

account as proof-of-principle, it may be good to provide some additional evidence, or at

least to improve reporting in terms of recording quality (e.g. unit isolation versus MUA,

recording stability, etc..).

Actually, while method describes some spectral analysis of ephys traces there is nothing

shown in results.

We found recordings to be stable over the

duration of a single session. However, the

number of units varied between recording days.

Shortly after the surgery (first recording

performed 8 days after surgery) the number of

units was relatively low and increased in the

second recording session. From this point

onwards we observed a continuous decline in the

number of units.6

Further details and a new figure (Fig. 7) were added to the manuscript (line 505):

"Units were well isolated (Fig. 7C) and the recordings were stable across the duration of

a session (Fig. 7A and B). Units B and C started firing when the animal started sleeping or

stopped moving inside the sleep-box (Fig. 7A). The number of units varied between

recording days. 8 days after the surgery the number of units was relatively low and

increased in the second recording session. From this point onwards, we observed a

continuous decline in the number of units, as expected in chronic targeting with fixed

probes.”

Other comments:

4) In reading a bit about the background of this paper, we came across this study

(https://www.pnas.org/content/111/52/18739; Low, Gu, and Tank 2014 PNAS). We feel it

is useful to show how others have addressed the challenge of this sinus physically

impeding implantation of midline structures. We encourage you to cite this paper together

with any other, to illustrate how various approaches have beed used and to discuss how

yours may benefit the implantation of large lenses for imaging medial structures.

Added in the introduction (line 69):

"This approach has recently been used to implant optical recording devices for

prefrontal cortex and medial entorhinal cortex imaging in head-fixed animals (Low, Gu, &

Tank 2014).”

Reference was also added in the reference list.

5) You claim this is a general procedure for rodents, but we wonder whether the SSS

morphology is similar in rats and mice? Given the interest of mice for the community we

feel it maybe useful to know more about the vascular morphology of the SSS and

associated branches in both species. For instance, is there an inferior sagittal sinus?

Added in the discussion (line 587):

"Since there is preservation of the dorsal venous system (SSS, transverse sinuses,

rostral and caudal rhinal veins) between rats and mice (Xiong et al., 2017) it seems

feasible that this method should be applicable to mice, with minimal adaptations.”

6) Resection involves only the anterior part of the SSS. Intervention at more posterior parts

may lead to different complications (the sinus is thicker there). Maybe useful to comment.

Added in the discussion (line 579):

"Also, since our surgical site was relatively anterior and we did not test the procedure at

more posterior coordinates where the SSS is thicker, we cannot claim feasibility across the

whole SSS extension. Nonetheless, at least one study employing a surgical procedure to 7

expose the pineal gland, located under the confluence of the SSS and the transverse

sinuses, reported no surgical complications and a comparably high success rate in the

removal of a more posterior section of SSS (Maganhin et al., 2009). This strongly suggests

that the procedure might be applicable in other sections of the SSS.”

7) Line 86: “..to partially transect..” why partial? The transection procedure looks like it

compromises the blood flow. Please, clarify

Corrected in the text.

8) In the methods, the stereotaxic coordinates for the tetrode implants were not provided.

Also, there is no description of the hyperdrive and tetrode dimensions. For instance, what

is the distance between the different tetrodes and the footprint dimension?

Are recordings bilateral or unilateral? Please, provide some additional details.

We implanted two animals with octrodes after SSS transection. Octrodes were used

due to their increased structural rigidity. However, recordings were performed in the

tetrode configuration.

The first animal served as a targeting test and was implanted with a bundle of octrodes

that were not connected to any recorded system. 10 Octrodes dipped in DiI stain were

carried inside polyamide tubes embedded in epoxy resin. One day after the surgery,

electrolytic lesions were performed under anesthesia and the animal was sacrificed.

Postmortem analysis revealed accurate bilateral targeting of SCN (Figure 6-1A).

The second animal was implanted with a functional Hyperdrive carrying 15 independent

octrodes for chronic freely moving recordings. Each octrode consisted of eight 12.7 μm

Nichrome wires (Sandvik). The distance between octrodes was approximately 166μm. A

single rectangular octrode bundle (1.46x1.27 mm, including the surrounding epoxy used to

fix the polyamide tubes) was implanted to bilaterally target hypothalamus at AP: -0.33 to -

1.08, ML: 0.5 to -0.5 and DV: -9.2 (Figure 6B). Once the dental acrylic hardened, the

octrodes were manually lowered to the desired depth and glued in place. Octrodes were

fixed throughout the recording experiment.

Although aiming at simultaneous bilateral recordings, postmortem histological

verification revealed unilateral targeting and final coordinates more posterior than

expected, due to an angled and misplaced bundle (see figure below). This was due to

unleveled drive and octrode insertion during the surgery rather than related to SSS transection. Check the attached figure for details on this mistargeting.8

Photographs of the retrieved octrode bundle and hyperdrive

Additional details were added to the results (line 486) and methods (line 155) sections.

9) Please, state whether or not the SHIRPA assessments were blinded.

Although the experimenter was not blind to the procedures, animals from the two

groups (sham and ST) were housed together and randomly chosen for assessment.

10) page 13, line 363, we suggest using the word “similar” instead of “comparable"

because one of us misread the sentence twice and thought it said that the ST rats shows a

preference for the novel arm “compared to” the sham rats.

Corrected in the text (line 401).

11) In figure 1F, it would be helpful adding something like a dotted line and errors showing

the location and direction of the dural incisions. This visual aid would complement the text

and help with understanding how to replicate the method.

Dotted lines and arrows were added to Figure 1F.9

12) Was the anesthesia isofluorane and also Ketamine/Medetomidine? Or was isofluorane

used to slightly sedate the animals before injecting the Ket/Med?

In our experience, Ketamine+Medetomidine was insufficient to keep the anesthesia

level throughout the procedure while also being an unreliable method to use in isolation,

as some animals died due to dosage reinforcements in previous prolonged surgeries in

the lab.

Therefore, we tested this experimental procedure and decided to induce anesthesia

with an intraperitoneal injection of Ketamine (37,5 mg/kg) + Medetomidine (0,5 mg/kg) and

use a volatile mixture of 0.5 to 1% isoflurane in oxygen throughout the procedure to

maintain anesthesia. Animal reflexes and breathing were monitored by the experimenter

throughout the surgery.

The detailed experimental protocol is described in Detailed Protocol (attached to this

letter).

13) Line 118: “.. no animal was lost during surgery” but then in discussion line 490: “From

a total of 26 frats only one animal died during surgery...”. Please check and clarify.

Corrected in the text (line 104).

14) Line 135: ...after a 5 minutes rest period” What does it mean? You mean 5 min of no

injection?

Changed in the text (line 134).

15) Methods: please clarify post-surgical treatment. Are analgesics or anti-immflamatory

treatment used? Any antibiotics? For how long?

Details were added to the methods section (line 168).

16) Methods of pathological analysis is the entire head cut in 4um sections. How is the

decalcified procedure? Please, specify these procedures with sufficient details.

Details were added to the methods section (line 236):

"10 weeks after SSS transection, rats were euthanized with an Isofluorane overdose

and immediately decapitated. Whole heads were immersion fixed in 10% neutral

buffered formalin, followed by 72 hours in a decalcifying solution (Surgipath

Decalcifier Leica), and then routinely processed for paraffin embedding, sectioned at 4

μm (15 to 20 sections distancing 100 μm), and stained with hematoxylin and eosin. 10

Lesions were classified by a veterinary pathologist blind to the experimental groups, and

scored according to the severity: absent, minimal, mild, moderate, marked, and severe. To

quantify the histological damage, lesioned areas were measured in five serial sections per

animal (across 1.5 mm), centered in the zone where the transection was performed, and

normalized to the whole slice area. All slides were digitally scanned using Hamamatsu

NanoZoomerSQ, and representative images were obtained using NDP.view2 software

(Hamamatsu).”

17) Regarding tetrode recordings: how clear is penetration and retraction in terms of

bleeding or any other damage? This may compromise microdrive movements and/or

probe recovery after experiment. Giving this is a major motivation for adopting this surgical

procedure it may be useful to know whether it solves this issue.

This hyperdrive was designed for chronic fixed targeting. Octrodes were not moved

throughout the duration of the experiment. Therefore, a small “ball” of super glue was

attached at the desired octrode length (above the upper end of the polyamide tubes) to

serve as a bumper, effectively allowing octrodes to be manually lowered and glued in their

final target depths, without further adjustments.

Penetrations were performed shortly after fixation of the drive to the animal’s skull,

precluding visual access to octrode penetration. This lowering movement was smooth and

unimpeded and we did not observe blood exiting the polyamide tubes, nor postmortem

evidence of extensive bleeding, namely at the surface of the brain or surrounding the

craniotomy.

The postmortem extraction of the probe was performed by ventrally exposing the brain

and gently freeing it from the surrounding skull. Octrodes were found to be straight (see

point 8) and the Hyperdrive was retrieved after careful removal of bone and dental acrylic

remains.

Additional details were added to the methods section (line 161 and 173).

18) Line 342: “... all timepoint tested” Only 4 and 8 week time points are tested. Check line

364 also.

Corrected in the text (line 380 and 402)

19) Lines 418 and 429: it should refer to Fig.5 and 6 not 4 and 5.

Corrected in the text.1112

20) Lines 508: access with an angle make the approach possible, even if not optimal, as

described in the introduction.

Although it is possible to target midline structures with angled approaches, this is not

viable for simultaneous bilateral recording, as most drive designs preclude assembling

bundles with lateral-to-midline converging angles. Our approach makes possible bilateral

targeting of midline structures by enabling direct vertical access. We do not feel we are

overstating, rather providing a conclusion based on our observations.
