# Supplementary figures and images for: Transection of the Superior Sagittal Sinus Enables Bilateral Access to the Rodent Midline Brain Structures
Source: eNeuro. 2021 Jul 14;8(4):ENEURO.0146-21.2021. doi: 10.1523/ENEURO.0146-21.2021 (PMC8281263; doi:10.1523/ENEURO.0146-21.2021)

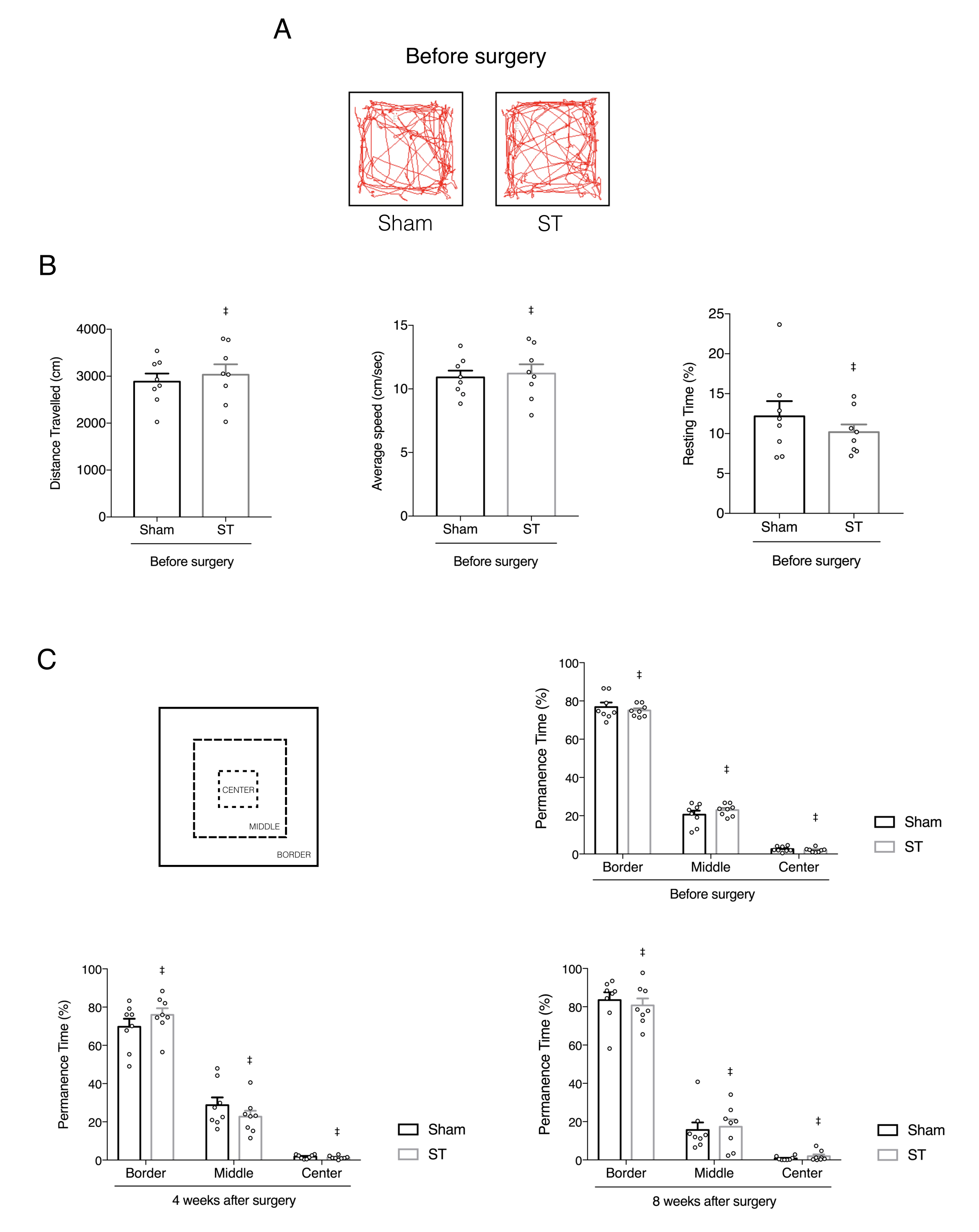

Supplement: Extended Data Figure 2-1 — ST rats exhibit locomotor and exploratory behavior similar to sham-operated animals in all time points tested. Locomotion and exploratory behavior were assessed by the open-field test before surgery and at four and eight weeks postsurgery. A, Representative trackplots of ST and sham-operated animals before surgery. B, No significant differences were found in total distance traveled, average speed and total resting time (n = 8, ‡p > 0.05, ST compared to sham, two-tailed unpaired t test) during presurgery testing. After basal assessment, animals were randomly assigned to experimental (ST) and control (sham) groups. Permanence in the open-field’s subregions was similar between groups in all time points tested (n = 8, ‡p > 0.05, ST compared to sham, two-way ANOVA followed by Tukey’s multiple comparisons post hoc test; C). All values are mean ± SEM. Download Figure 2-1, TIF file. [file enu-eN-MNT-0146-21-s03.tif]

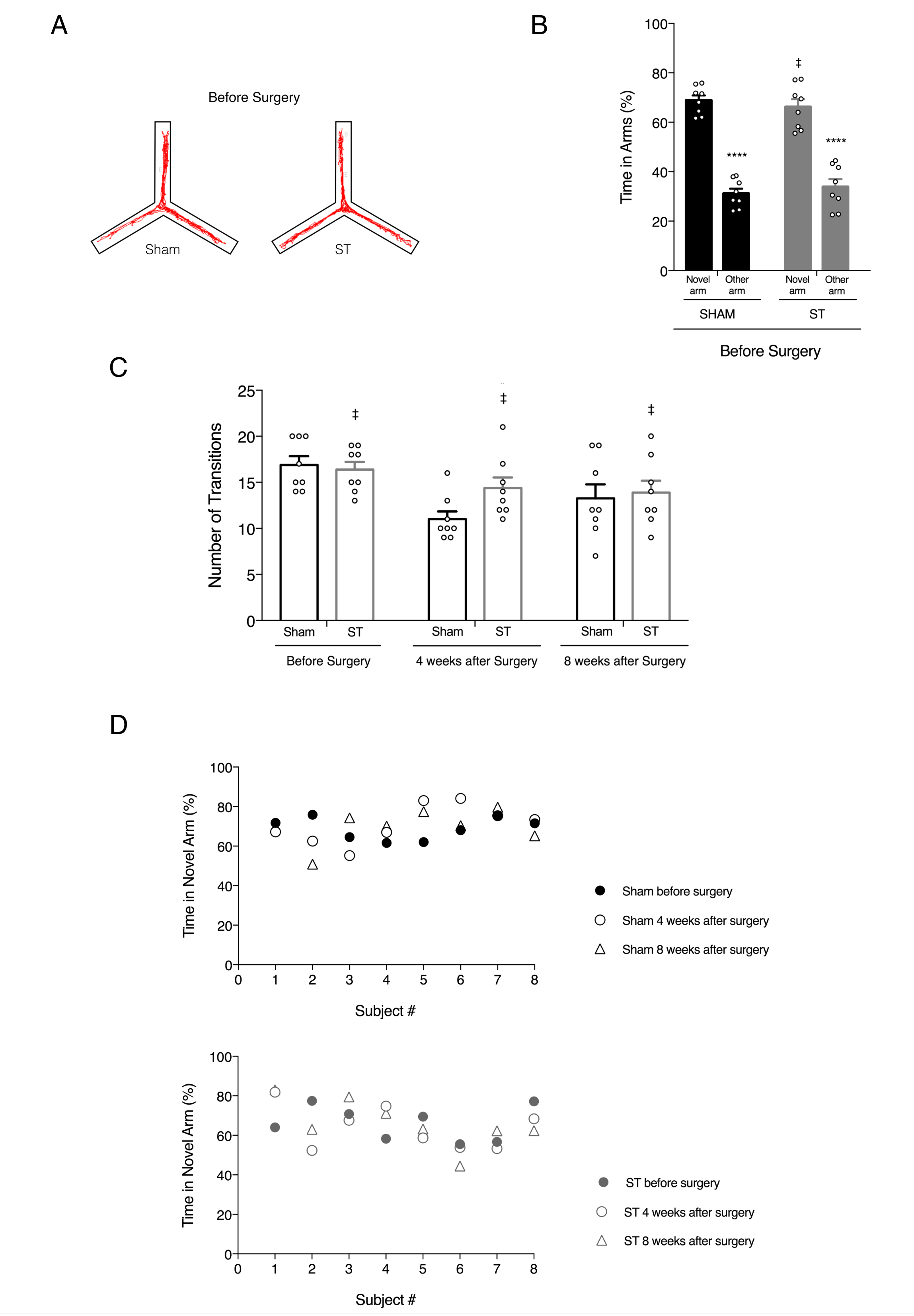

Supplement: Extended Data Figure 3-1 — Short-term spatial memory performance of ST rats is similar to sham-operated animals in all time points tested. Spatial memory performance was assessed by the Y-maze test before surgery and at four and eight weeks postsurgery. A, Representative trackplots of ST and sham-operated animals before surgery. B, Quantification of the time spent by sham and ST animals in novel versus other arm at baseline assessment. Both groups showed preference for the novel arm (n = 8, means ± SEM, ****p < 0.0001, novel arm compared to other arm, two-way ANOVA followed by Tukey’s multiple comparisons post hoc test). After basal assessment, animals were randomly assigned to experimental (ST) and control (sham) groups. C, Quantification of the number of transitions between arms. No differences were found between groups (n = 8, means ± SEM, ‡p > 0.05, ST compared to sham, repeated measures two-way ANOVA followed by Bonferroni’s multiple comparisons post hoc test). D, Quantification of the time spent by the same animal in the novel arm at different assessments. Download Figure 3-1, TIF file. [file enu-eN-MNT-0146-21-s04.tif]

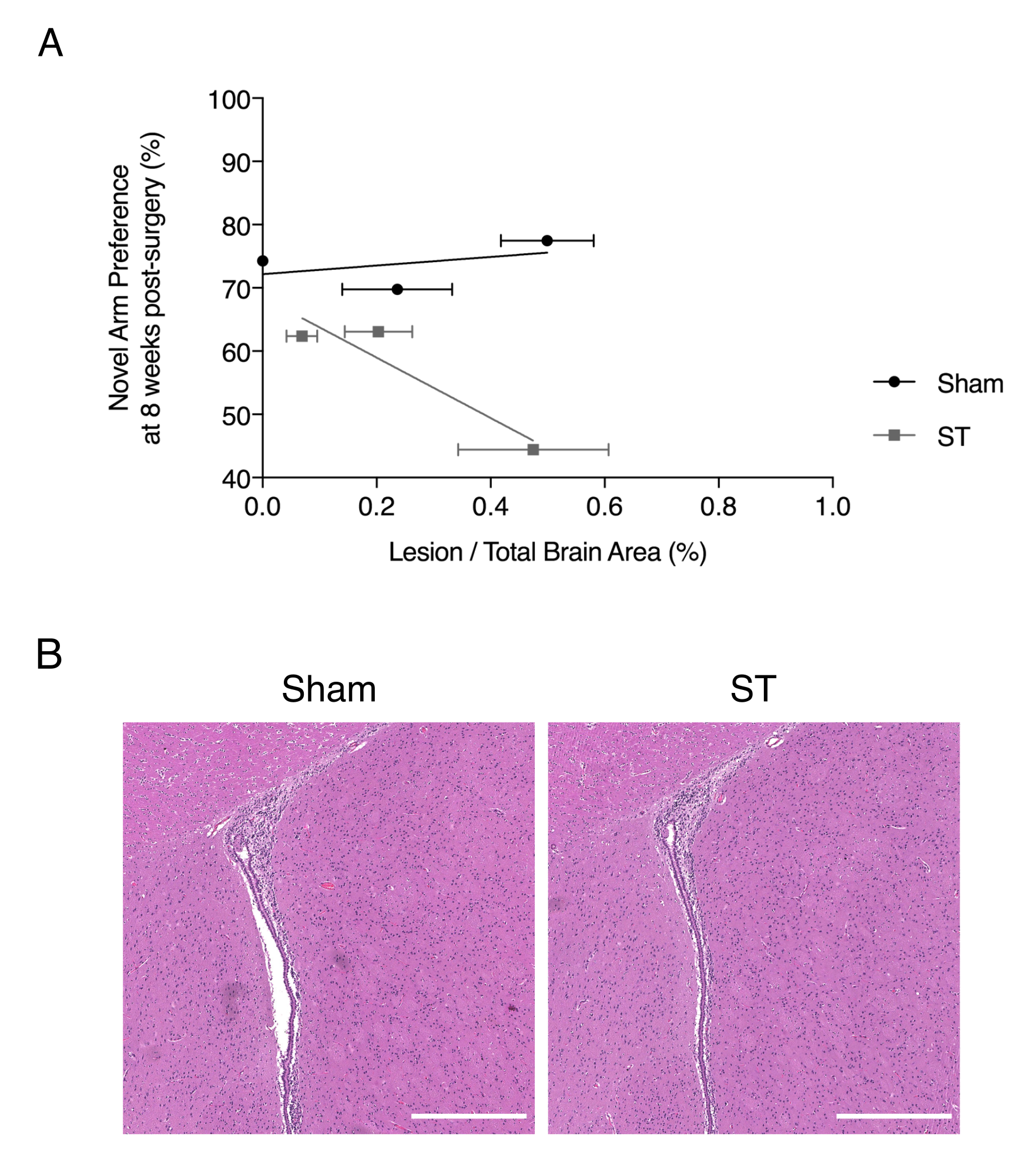

Supplement: Extended Data Figure 4-1 — Histological damage is not correlated with performance and did not involve ventricular alterations. A, The extent of damage was quantified in three animals from each group, by measuring the lesioned area in five serial sections per animal (across 1.5 mm) and normalizing to the whole-brain area. No correlation was found between histological damage and Y-maze performance at eight weeks postsurgery (sham: r2 = 0.19, p = 0.71; ST: r2 = 0.87, p = 0.23; Pearson’s correlation). No significant differences were found in histological damage between groups (n = 3, p > 0.05, ST compared to sham, two-tailed unpaired t test). All values are mean ± SEM. B, Normodimensioned lateral ventricles of sham and ST animals. Scale bar: 500 μm. Download Figure 4-1, TIF file. [file enu-eN-MNT-0146-21-s05.tif]

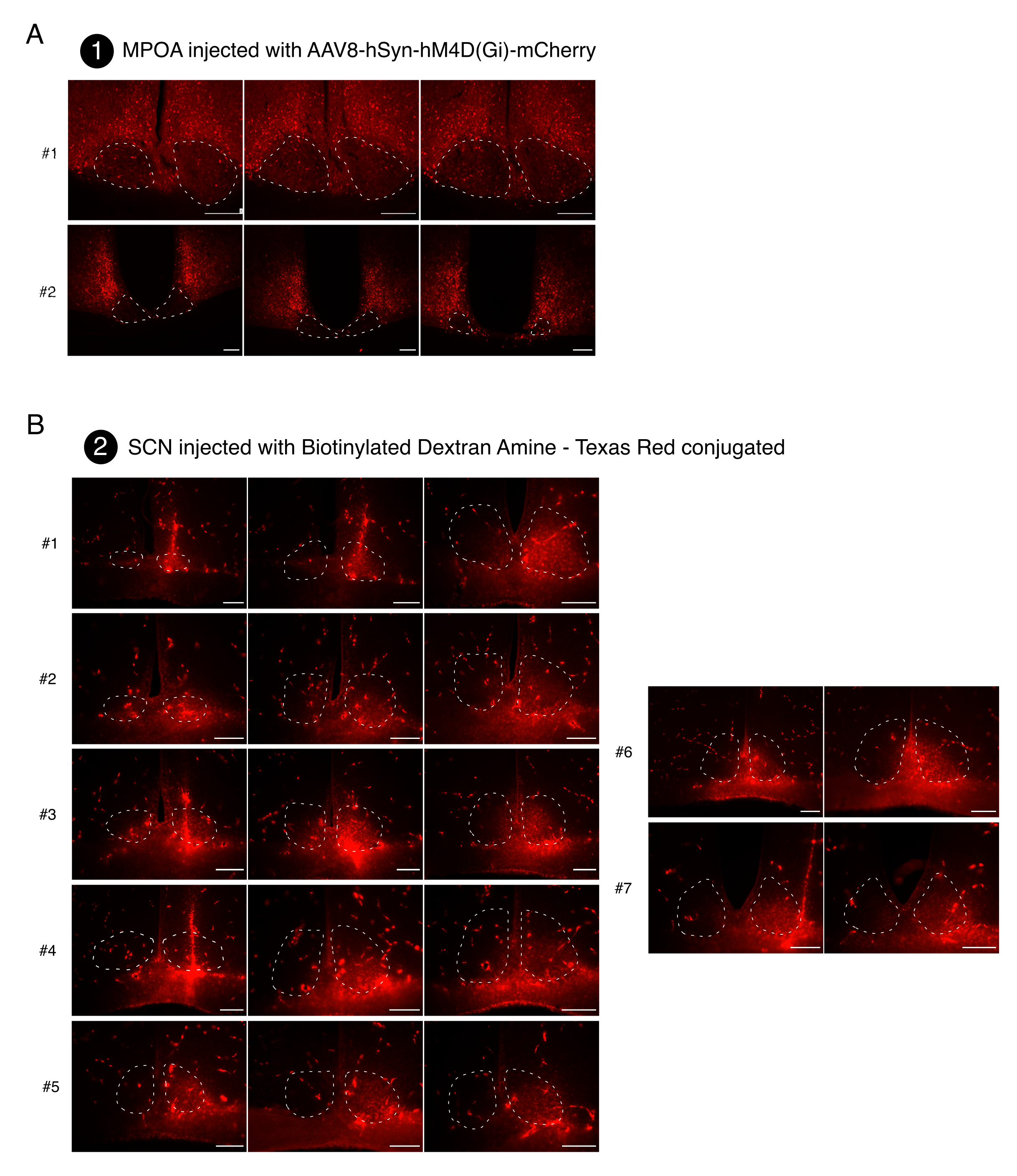

Supplement: Extended Data Figure 5-1 — SSS transection allows consistent microinjection of deep hypothalamic nuclei. Photomicrograph of coronal sections showing extensive AAV8-hSyn-hM4D(Gi)-mCherry expression in the MPOA at five weeks postinfection (1) and strong BDA–Texas Red uptake in the SCN at 11 d postinjection (2). SSS allows consistent targeting of MPOA and SCN across subjects. Scale bar: 250 μm. Download Figure 5-1, TIF file. [file enu-eN-MNT-0146-21-s06.tif]

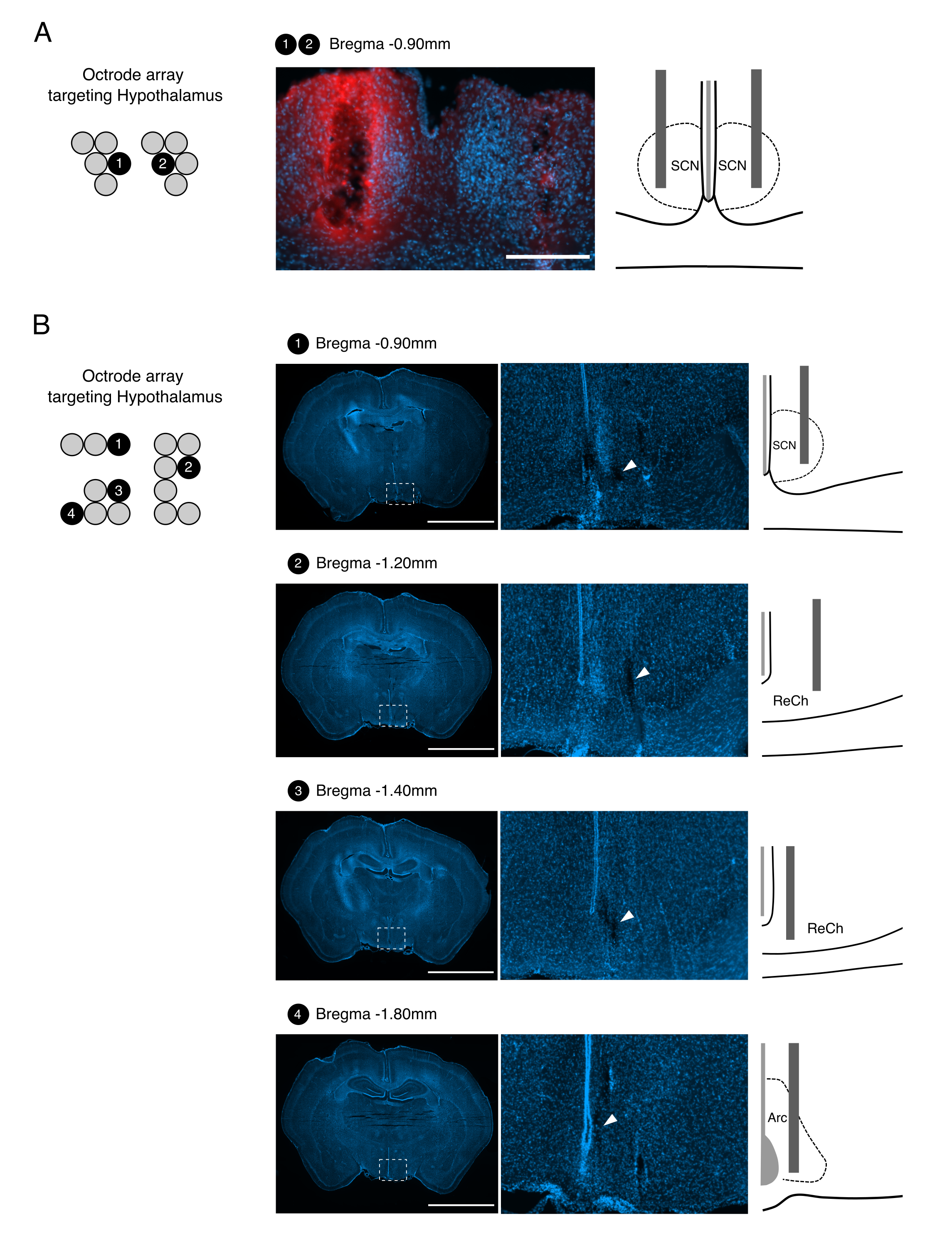

Supplement: Extended Data Figure 6-1 — SSS transection allows multisite recording from deep midline brain structures. A, Postmortem analysis revealed accurate bilateral targeting of SCN in preliminary targeting test, as indicated by the electrolytic lesion and DiI staining. Cell nuclei are stained with Hoechst in blue fluorescence. Octrode tips were dipped in DiI stain (red fluorescence). Scale bar: 250 μm. B, Histological verification of octrode tracks confirms targeting of SCN, ReCh, and Arc. Cell nuclei are stained with Hoechst in blue fluorescence. White arrows indicate octrode tips. Scale bar: 0.50 cm. Download Figure 6-1, TIF file. [file enu-eN-MNT-0146-21-s07.tif]
